# New Infestin-4 Mutants with Increased Selectivity against Factor XIIa

**DOI:** 10.1371/journal.pone.0144940

**Published:** 2015-12-15

**Authors:** Vladimir N. Kolyadko, Sofya V. Lushchekina, Tatiana A. Vuimo, Stepan S. Surov, Ruzanna A. Ovsepyan, Vera A. Korneeva, Ivan I. Vorobiev, Nadezhda A. Orlova, Leonid Minakhin, Konstantin Kuznedelov, Konstantin V. Severinov, Fazoil I. Ataullakhanov, Mikhail A. Panteleev

**Affiliations:** 1 Center for Theoretical Problems of Physicochemical Pharmacology of Russian Academy of Sciences, Moscow, Russian Federation; 2 Emanuel Institute of Biochemical Physics of Russian Academy of Sciences, Moscow, Russian Federation; 3 Federal Research and Clinical Center of Pediatric Hematology, Oncology, and Immunology named after Dmitry Rogachev of Ministry of Health, Moscow, Russian Federation; 4 Center “Bioengineering” of Russian Academy of Sciences, Moscow, Russian Federation; 5 Waksman Institute of Microbiology, Rutgers The State University of New Jersey, Piscataway, New Jersey, United States of America; 6 Department of Physics, Lomonosov Moscow State University, Moscow, Russian Federation; 7 Faculty of Biological and Medical Physics, Moscow Institute of Physics and Technology, Dolgoprudny, Russian Federation; 8 HemaCore LLC., Moscow, Russian Federation; Russian Academy of Sciences, Institute for Biological Instrumentation, RUSSIAN FEDERATION

## Abstract

Factor XIIa (fXIIa) is a serine protease that triggers the coagulation contact pathway and plays a role in thrombosis. Because it interferes with coagulation testing, the need to inhibit fXIIa exists in many cases. Infestin-4 (Inf4) is a Kazal-type inhibitor of fXIIa. Its specificity for fXIIa can be enhanced by point mutations in the protease-binding loop. We attempted to adapt Inf4 for the selective repression of the contact pathway under various *in vitro* conditions, e.g., during blood collection and in ‘global’ assays of tissue factor (TF)-dependent coagulation. First, we designed a set of new Inf4 mutants that, in contrast to wt-Inf4, had stabilized canonical conformations during molecular dynamics simulation. Off-target activities against factor Xa (fXa), plasmin, and other coagulation proteases were either reduced or eliminated in these recombinant mutants, as demonstrated by chromogenic assays. Interactions with fXIIa and fXa were also analyzed using protein-protein docking. Next, Mutant B, one of the most potent mutants (its *K*_*i*_ for fXIIa is 0.7 nM) was tested in plasma. At concentrations 5–20 μM, this mutant delayed the contact-activated generation of thrombin, as well as clotting in thromboelastography and thrombodynamics assays. In these assays, Mutant B did not affect coagulation initiated by TF, thus demonstrating sufficient selectivity and its potential practical significance as a reagent for coagulation diagnostics.

## Introduction

Coagulation factor XIIa (fXIIa) auto-activates upon binding to negatively charged surfaces (e.g., activated platelets or the bacterial cell wall). This process is called “contact activation” and is amplified by plasma kallikrein; it triggers the coagulation cascade via factors XIa (fXIa) and IXa (fIXa) [1,2]. Contact activation was found to be a key element in thrombosis development [3,4]. Knockout or inhibition of fXIIa resulted in reduced mortality and thrombus weight in a number of animal models, though hemostasis remained intact in these animals [5,6]. Additionally, contact activation is responsible for clot formation when blood is manipulated *in vitro* or *ex vivo*, e.g., when using a cardio-pulmonary bypass [7], collecting blood, and assaying global coagulation, activated partial thromboplastin time (aPTT) or activated clotting time (ACT). Additionally, *in vitro* assays of coagulation triggered by tissue factor (TF) (thrombin generation, thromboelastography, thrombodynamics, and flow chamber assays) suffer from artifacts caused by contact activation [8]. To date, only corn trypsin inhibitor (CTI) has been applied to inhibit fXIIa in various assays [9], however, a recent re-examination of its selectivity has shown off-target activity against fXIa and other proteases [10]. Hence, a highly efficient and selective inhibitor of fXIIa would be a valuable reagent for *in vitro* diagnostics and *in vivo* plasmapheresis systems [11].

Infestin-4 (Inf4) is the 7th C-terminal domain of the infestin protein whose cDNA was extracted from the salivary glands of the blood-sucking insect *Triatoma infestans* [12,13]. Wild-type infestin-4 (wt-Inf4), a 56 amino acid Kazal-type protein, is a canonical inhibitor and has the reactive site sequence P^2^-FRNYVPV-P^5’^ (nomenclature of Schechter and Berger [14]), where P^1^ Arg10 –P^1’^ Asn11 is a scissile bond. Wt-Inf4 inhibits fXIIa (with a *K*_*i*_ = 0.1 nM), as well as trypsin (*K*_*i*_ = 11 nM), plasmin (*K*_*i*_ = 2.1 nM), and fXa (*K*_*i*_ = 53 nM) [13]. Recently, a wide-ranging evaluation of Inf4 potency as an anti-thrombotic substance was carried out in a number of pre-clinical settings, including the inhibition of fXIIa activity towards chromogenic and physiological substrates; the profiling of selectivity against a set of coagulation proteases from humans, rats, and rabbit; the repression of contact-activated thrombin generation in plasma; and the down-regulation of *in vivo* thrombus growth [15]. In the latter study, it was shown that the off-target activity against fXa caused a 1.5-fold increase in bleeding tendency, emphasizing a need to enhance the selectivity of Inf4. An attempt to increase Inf4 selectivity for fXIIa was made using a phage-display selection of the protease-binding loop sequences [16]. Inf4 variants that bound fXIIa contained Ser, Thr, or Asn amino acid residues at the 9th position (P^2^ position of the reactive site); at the 11th position (P^1’^), Arg or, less frequently, Asn was found. The authors selected the mutant Inf4-Mut15 with the P^2^ –P^5’^ sequence TRRFVAV that inhibited neither fXa nor plasmin [16]. However, the reactivity of this mutant towards other coagulation proteases has not been reported. Moreover, this mutant has not been tested in plasma, i.e., there was no indication of its impact on the coagulation system. Furthermore, the mechanism responsible for the increased selectivity remains unclear.

The purpose of this study was to investigate and improve the potency of infestin-4 as a reagent to repress the contact pathway in a number of *in vitro* settings. A new set of Inf4 mutants with no or reduced off-target activities was designed and tested in a wide range of global coagulation assays; as a result, Mutant B was selected as the most selective mutant of Inf4.

## Materials and Methods

### Reagents and materials

The following materials were obtained from the indicated sources: human fXIa, fIXa, fXa, thrombin, plasma kallikrein, and activated protein C (aPC) (Hematologic Technologies; Essex Junction, VT); recombinant tissue plasminogen activator (tPA) Alteplase® (Genentech; South San Francisco, CA); human α-fXIIa (Enzyme Research Laboratories; South Bend, IL); recombinant factor VIIa (fVIIa) Novoseven® (Novo Nordisk; Bagsværd, Denmark); Lys-plasmin and chromogenic substrates Spectrozyme FVIIa, Spectrozyme FIXa, Spectrozyme PCa, Spectrozyme tPA (Sekisui Diagnostics; Stamford, CT); S-2302, S-2366, S-2765 (Chromogenix; Milano, Italy); and Pefachrome TH 5244 (Pentapharm; Basel, Switzerland). CTI provided by Gamma (Pushchino, Russia) was extracted from corn, purified with ammonium sulfate, acetone precipitation, trypsin-affinity and ion-exchange chromatography, as described previously [17]. *Luffa cylindrica* trypsin inhibitor (LCTI-III) was chemically synthesized (Creative Dynamics; Shirley, NY). All other reagents were from (Sigma-Aldrich; St. Louis, MO) unless otherwise noted.

### Expression of the thioredoxin-fused infestin-4 and its mutants

An infestin-4 gene coding for 56 amino acids was synthesized by the PCR fusion of five primers. The oligonucleotides AS-KAZ-1F and AS-KAZ-2R with 16-nt-long complementary 3’-termini (S1 Table), as well as AS-KAZ-3F and AS-KAZ-4R (18-nt-long complementary ends), were annealed and extended, resulting in two DNA fragments (105 bp and 115 bp, respectively). Because AS-KAZ-2R and AS-KAZ-3F had 17-nt-long overlapping 5’-termini, the 105-bp- and 115-bp-long products were fused and extended. A final DNA product (214 bp) was generated by the extension of the 3’-terminus using the primer AS-KAZ-5R. This product was amplified, cloned into the p10E plasmid using NheI and HindIII restriction sites and subcloned using NcoI and HindIII (Fermentas; Vilnius, Lithuania) into the pET32a plasmid (Merck; Darmstadt, Germany), which encodes thioredoxin I (Trx) and the thrombin cleavage site. The pET32-Inf4 plasmid was transformed into *Escherichia coli* BL21 (DE3) cells via heat-shock, and selected clones were grown in TB medium with 0.1 mg/ml ampicillin and 0.1% glucose. Expression of Trx-Inf4 (GenBank KJ183178) was induced with 1 mM IPTG for 3 hours at 30°C. The cells were harvested and resuspended in 10 mM Tris-HCl pH 7.5, 0.1% Triton-X100, and 10 μg/ml lysozyme, and then, the cells were incubated on ice for 30 min and sonicated. The clarified lysate was supplemented with 40 mM Tris-HCl pH 7.5, 500 mM NaCl, and 10 mM imidazole and applied at 10 ml/min on a Chelating Sepharose Fast Flow column (GE Healthcare; Little Chalfont, UK). Weakly bound proteins were eluted with a solution containing 50 mM Tris, pH 7.5, 500 mM NaCl, and 100 mM imidazole, followed by a 50 mM Tris-HCl pH 7.5 solution. Trx-Inf4 was eluted with 50 mM Tris-HCl pH 7.5, 500 mM imidazole solution and applied to a Tricorn 10/100 column (GE Healthcare) with SOURCE 30Q strong anion-exchange resin in 20 mM Tris, pH 8.0. Monomeric Trx-Inf4 was eluted with a linear NaCl gradient from 0 to 500 mM at flow rate of 8 ml/min. Trx-Inf4 (150 mM NaCl) was collected and concentrated by ultrafiltration with Vivaspin 6 PES 5 kDa (Sartorius; Goettingen, Germany) to 10–14 mg/ml. The protein concentration was determined by UV-spectroscopy. The pure Trx-Inf4 yield was approximately 60 mg per liter of cell culture.

To substitute the reactive site residues Phe9, Asn11, Tyr12, and Pro14 in wt-Inf4 and generate the Trx-fused Mutant 15 (GenBank KJ183179), Mutant A (GenBank KJ183180), Mutant B (GenBank KJ183181), and Mutant C (GenBank KJ183182), we used PCR mutagenesis from the pET32-Inf4 template, essentially as described in [18]. This procedure utilized two vector-specific primers and two specific oligonucleotides that had 30-nt complementary sequences at their 5’-termini; these overlapping parts of the oligonucleotides incorporated the designed mutations. One DNA fragment (165 bp) was amplified with a vector-specific forward primer (VectorFor) and a mutation-specific reverse primer (MutRev); the second fragment (181 bp) was produced by PCR with a mutation-specific forward primer (MutFor) and a vector-specific reverse primer (VectorRev) (S1 Table). The generated fragments were combined and fused by PCR using a pair of vector-specific primers. The resulting DNA fragment (320 bp) was cloned into pET32a using the NcoI and HindIII sites (the NcoI recognition site was located 20 bp downstream of the VectorFor sequence in the plasmid). The plasmids pET32-MutA, -MutB, -MutC, and–Mut15 were transformed into *E*. *coli* BL21 (DE3) cells. The cells were plated on ampicillin-containing medium, and clones were selected by PCR screening and were confirmed by sequencing. The mutant proteins were expressed and purified as described above for wild-type infestin.

Global coagulation assays were performed using Mutant B that was preliminarily cleaved from Trx with 1 U of bovine thrombin (MP Biomedicals; Santa Ana, CA) per 1 mg of the fusion protein. The cleaved Mutant B protein (the reaction product) was purified from both thrombin and Trx. The reaction mixture was applied on a SP Sepharose HiTrap column (GE Healthcare) at 0.5 ml/min followed by washing the column with 20 mM Tris-HCl, pH 8.0. The target product was collected in the flow-through fraction which was then applied to a Tricorn 10/100 column containing SOURCE 15Q resin (GE Healthcare). The residual thrombin level in the Mutant B preparations was below the limit of detection using the thrombin-specific chromogenic substrate. The total yield of Mutant B (13 kDa) was 25 mg per liter of cell culture.

### CMTI-III expression

The most efficient system for the expression of the recombinant *Cucurbita maxima* trypsin inhibitor CMTI-III (29 aa) was a fusion with the polyhistidine (His)-tagged domain B1 of the *Streptococcus sp*. protein G (GB1) [19]. Other versions, including 1) His-tagged CMTI-III, 2) a polypeptide containing several CMTI-III domains, 3) fusion with glutathione-S-transferase (pGEX-4T-1; GE Healthcare), and 4) fusion with the chitin-binding domain (pTYB1 intein system; New England Biolabs; Ipswich, MA), were less favourable because of either protein degradation, misfolding, or low yield [20]. A DNA fragment coding for GB1, a linker peptide, and CMTI-III [21] was constructed from four oligonucleotides with 20-nt-long complementary termini (S1 Table). Two primer pairs (FuCMTI1up and FuCMTI2low; FuCMTI3up and FuCMTI4low) were annealed and extended with PCR. The reaction products FuCMTI1up-FuCMTI2low (180 bp) and FuCMTI3up-FuCMTI4low (175 bp) were annealed and extended because the primers FuCMTI2low and FuCMTI3up had 20-nt-long complementary 5’-termini. The final DNA fragment (335 bp) was cloned into pET28a (Merck) with the NcoI and EcoRI sites (New England Biolabs) that were introduced by the FuCMTI1up and FuCMTI4low primers, respectively. The generated plasmid p28FuCMTI was transformed into *E*. *coli* NovaBlue competent cells, sequenced and transformed into *E*. *coli* BL21 (DE3) cells, which were grown in LB with 0.025 mg/ml kanamycin. The expression of GB1-CMTI-III (GenBank KJ183183) was induced overnight with 0.4 mM IPTG at 18°C, and the protein was purified on Ni-NTA resin, with a yield of 2.5 mg CMTI-III per liter of cell culture.

### Chromogenic assay

The selectivity profiles of the inhibitors were studied against ten major coagulation-related serine proteases. The respective protease (50 μl) was mixed with varying concentrations of an inhibitor (0.5 nM – 50 μM; 50 μl) in a buffered solution (100 mM Tris-HCl pH 7.4, 260 mM NaCl, 1.0% BSA) and incubated in a 96-well microtiter plate (Corning; Corning, NY) for 15 min at 37°C. An appropriate chromogenic substrate (100 μl) diluted with water was added to the mixture, and the kinetics of the p-nitroaniline (pNA) generation were monitored by measuring the absorbance at 405 nm using a Sunrise Microplate Reader (Tecan; Männedorf, Switzerland). The final concentration of each protease and its substrate in a total volume of 200 μl are listed below (Table 1). The initial rate of substrate hydrolysis (determined as a slope of the pNA generation curve) was normalized to the value at a zero inhibitor concentration and plotted versus the inhibitor concentration. This curve was fitted with the equation *V(%) = 100%/(1 + I/IC*_*50*_ *)*, where *I* is the inhibitor concentration, and *IC*_*50*_ is the inhibitor concentration that inhibited the protease amidolytic activity by 2-fold. The inhibitory constant *K*_*i*_ was calculated according to the Cheng-Prusoff equation *K*_*i*_ *= IC*_*50*_ */(1 + S/K*_*m*_ *)* [22], where *S* is the initial substrate concentration and *K*_*m*_ is the Michaelis constant (Table 1). The SD value for the inhibitory constant *K*_*i*_ accounted for both errors of measurement for *IC*_*50*_ and *K*_*m*_ and was calculated as the square root of the sum *(ΔIC*_*50*_ */(1 + S/K*_*m*_ *))* ^*2*^ + n*(K_*i*_ */(1 + K*_*m*_ */S)* *ΔK_*m*_ */K*_*m*_ *)* ^*2*^, where *ΔIC*_*50*_ –SD value for *IC*_*50*_, *ΔK*_*m*_ and *n*–SE for the *K*_*m*_ value and a number of measurements of this value, respectively.

**Table 1:** Coagulation-related proteases and their corresponding substrates used in the chromogenic assays. Concentrations of the serine proteases (nM) and their substrates (mM) in a final volume of 200 μl. The *K*_*m*_ values (mM) are expressed as the mean ± SE (number of repeats).

Protease	Protease concentration (nM)	Chromogenic substrate	Substrate concentration (mM)	*K*_*m*_ (mM)
XIIa	1	S-2302	0.2	0.18 ±0.03 (n = 4)
XIa	0.1	S-2366	0.5	0.93 ±0.20 (n = 4)
IXa	200	Spectrozyme FIXa	0.5	0.192 ±0.013 (n = 4)
Xa	0.5	S-2765	0.5	0.29 ±0.08 (n = 4)
thrombin	0.2	Pefachrome TH 5244	0.02	0.024 ±0.005 (n = 7)
VIIa	200	Spectrozyme FVIIa	0.5	0.86 ±0.39 (n = 2)
aPC	1	Spectrozyme PCa	0.2	0.24 ±0.06 (n = 4)
kallikrein	0.5	S-2302	0.25	0.23 ±0.04 (n = 5)
tPA	2	Spectrozyme tPA	0.2	0.40 ±0.07 (n = 4)
plasmin	2	S-2366	0.4	0.56 ±0.18 (n = 3)

Measurement of the *K*_*m*_ values was performed essentially as described above. The appropriate protease (100 μl) was mixed with varying concentrations (approx. 0.5*K_*m*_− 3*K_*m*_) of the appropriate chromogenic substrate (100 μl) in the indicated buffer. Kinetics of the pNA generation were monitored immediately at 37°C. The substrate cleavage rate was plotted versus the concentration of substrate and fit with the Michaelis-Menten equation; the *K*_*m*_ value was calculated as the mean ± SE.

### Molecular modeling

#### Model set up

Seven models of the fXIIa inhibitors (infestin-4, four mutants, CMTI-III, and LCTI-III) were prepared for the following molecular dynamics (MD) simulations. Five models of wt-Inf4 and its mutants were constructed based on the X-ray structure of wt-Inf4 (PDB ID 2ERW, resolution 1.40 Å [16]) with the amino acids manually substituted. The first three residues of the infestin-4 protein, which missing in the X-ray structure, were rebuilt using the *psfgen* module of VMD [23]. The CMTI-I crystal structure (PDB ID 1LU0, at a resolution 1.03 Å [24]) was used to obtain the models of squash-type inhibitors; in the model of CMTI-III, the substitutions L8M and E9K were manually performed, while the LCTI-III model was constructed via a manual substitution of the V2I, L8M, K11S, K12S, V21I, H25N, and Y27F residues. The resulting 7 models were uniformly treated as follows: crystallographic water molecules found in the original X-ray structures were kept and more water molecules were added to form a rectangular box with a minimum distance of 10 Å between the protein molecule and the box edge. Na^+^ and Cl^-^ ions were added at a concentration of 0.15 M to make the systems electroneutral.

#### Molecular dynamics simulation

All models were then subjected to the MD simulations performed using NAMD 2.9 [25] software, a CHARMM36 force field [26], a TIP3P water model, a temperature of 298K, a pressure of 1 atm (NPT ensemble), and periodic boundary conditions at the Lomonosov supercomputer [27]. The systems were initially energy minimized for 1,000 steps; afterwards, the solvent was equilibrated for 1 ns with the protein atoms fixed, except for the mutated residues. The pre-equilibrated systems were fully energy minimized in 5,000 steps, followed by a 150-ns-long productive MD simulation run at 298K. These 150 ns trajectories were used to analyze the conformational changes and intramolecular interactions in the inhibitor molecules using custom written VMD-based tools. To generate the stabilized model structures of the inhibitors, the MD simulations were run at 298K followed by a cool down from 298K to 4K for 30 ns. These generated inhibitor models were used at the subsequent step of the protein-protein docking to the models of the coagulation proteases.

#### Protein-protein docking

We used a homology model structure of fXIIa that was previously built [10] because no X-ray structure for fXIIa in the active conformation was currently available. The homology model was based on the crystal structure of hepatocyte growth factor activator (PDB ID 2R0L, resolution 2.2 Å [28]) (47% similarity); it was docked with the models of wt-Inf4, its mutants, and the squash-type inhibitors obtained by the MD simulation (see above). In addition, structures of the inhibitors were docked to the crystal structure of fXa (PDB ID 2JKH, resolution 1.25 Å [29]).

Protein-protein docking was initially performed using three Web servers: ClusPro [30], Rosetta [31] and pyDock [32]. The constraints matching the P^1^ and P^1’^ inhibitor residues with the protease catalytic Ser and His were applied in ClusPro and pyDock. A relative position of the inhibitor-protease complex generated by ClusPro was set as an initial position in Rosetta. However, in a majority of the complexes generated by pyDock, the inhibitor was located distantly from the protease active site. Additionally, in many cases of flexible docking by Rosetta, the catalytic residues of the protease had moved too far from each other, which was an obvious artifact because such a conformation did not correspond to the active protease. The results obtained by ClusPro were the most consistent and are reported here. Molecular graphics were created using VMD [23] and PyMOL [33].

### Blood collection and plasma preparation

This study including the blood collection procedures was approved by the institution’s Ethics Committee of the Center for Theoretical Problems of Physicochemical Pharmacology (Moscow, Russia) and was performed in accordance with the Declaration of Helsinki. Blood (9 ml) was collected from healthy volunteers who provided written consent. In the coagulation experiments, normal plasma was prepared from citrated blood drawn from the median cubital vein into Vacuette plastic tubes with 3.2% sodium citrate (Greiner Bio-One; Kremsmünster, Austria). Platelet poor plasma (PPP) was prepared by centrifugation of the citrated blood at 1,600 *g* for 15 min. Platelet free plasma (PFP) was obtained from PPP by a second centrifugation at 10,000 *g* for 5 min. Normal PPP and PFP from 5–10 healthy donors were pooled and frozen at -80°C. A sample of frozen plasma was thawed in a 37°C water bath for at least 30 min prior to use. In the experiments with coagulation factor-deficient or -depleted plasma, we used lyophilized fXII-depleted plasma and lyophilized fVIII- and fIX-deficient plasma (Renam; Moscow, Russia), which were reconstituted with water according to the manufacturer’s instructions and clarified by centrifugation at 10,000 *g* for 15 min.

When clotting time of the uncitrated whole blood was measured, 0.7 ml of fresh whole blood drawn using a Vacuette 21G needle (Greiner Bio-One) immediately after the collection procedure was placed into a 1.5-ml polypropylene tube (Axygen; Union City, CA) pre-filled with 78 μl of fXIIa inhibitor or a vehicle (30 mM Hepes pH 7.4). The tubes were rotated at 10 rpm, and the time of blood clotting was visually examined.

### APTT and PT assays

The activity of the fXIIa inhibitors in plasma was estimated by their effects on the activated partial thromboplastin time (aPTT) and prothrombin time (PT). These assays were conducted according to standard protocols from the reagent manufacturer (Renam) using a Helena C-2 coagulometer (Helena Biosciences Europe; Gateshead, UK). Briefly, 50 μl of normal frozen-thawed PFP was incubated in the absence or presence of increasing concentrations of inhibitor (5.6 μl) at 37°C for 15 min; then, two equal aliquots of this mixture (25 μl each) were placed in two cuvettes (Helena Biosciences Europe) in the coagulometer. In the aPTT assay, a kaolin-cephalin mixture (25 μl) was added to each cuvette and incubated with plasma for 3 min to activate the contact pathway. The aPTT measurement was performed in duplicate and initiated by the addition of 25 mM CaCl_2_ (25 μl). Each experiment was performed at least 3 times. In the PT assay, a mixture of rabbit brain TF and 12.5 mM CaCl_2_ was added to plasma (50 μl in each cuvette) to initiate clotting. The anticoagulant activity of the inhibitor (designated as *CT*_*3*_) was quantitated as the concentration required to prolong the clotting time in the aPTT assay by 3-fold compared to normal plasma without inhibitor. *CT*_*3*_ was calculated by fitting the aPTT dose-dependent curve (fold increase) with a linear function, *P*_*1*_ *I+P_*2*_ (where *I* is an inhibitor concentration in μM, *P*_*1*_, *P*_*2*_ –coefficients, and Δ*P*_*1*_ and Δ*P*_*2*_ are the corresponding errors). *CT*_*3*_ was calculated as *(3 –P*_*2*_ *)/P*_*1*_. The standard error was the square root of *(ΔP*_*2*_ */P*_*1*_ *)* ^*2*^ *+ (ΔP*_1_*P_*2*_ */P*_*1*_^*2*^ *)* ^*2*^.

### Thrombin generation assay

The thrombin generation experiments were carried out essentially as previously described [34]. The thrombin fluorogenic substrate Z-Gly-Gly-Arg-AMC (Bachem; Bubendorf, Switzerland—740 μl of a 2.5 mM solution dissolved in buffer 18 mM Hepes pH 7.5, 130 mM NaCl, 10% DMSO) was added to a sample of frozen-thawed PPP (2960 μl). This mixture was incubated at 37°C with various concentrations of Mutant B, CTI, or a vehicle (30 mM Hepes pH 7.4), followed by the addition of 100 μl of this mixture into a 96-well microtiter plate. Thrombin generation was initiated by the addition of a trigger solution (20 μl), which contained CaCl_2_ (16.7 mM; final concentration in a total volume of 120 μl) and phospholipids (4 μM; phosphatidylcholine: phosphatidylserine 80:20). Phospholipid vesicles were prepared as previously described [35]. For the contact-activated generation, the trigger solution also included kaolin (kaolin was 200 times more dilute in a total volume of 120 μl than in the standard aPTT assay), while for the TF-induced generation, the trigger solution included rabbit TF (Renam; final concentration 5 pM). The kinetics of the 7-amino-4-methyl-coumarin (AMC) release were continuously measured immediately after the addition of the trigger solution at 37°C for 60 min using an Appliskan Multimode Microplate Reader (Thermo Scientific; Helsinki, Finland) (excitation and emission wavelengths: λ_exc_ 355 nm, λ_em_ 460 nm). In parallel, the fluorescence was measured in calibration wells containing the plasma-substrate mixture (100 μl) and 20 μl of AMC (a final concentration 8 μM in a total volume of 120 μl). The background fluorescence level was measured in wells containing the plasma-substrate mixture (100 μl) and 20 mM Hepes pH 7.5, 145 mM NaCl (20 μl). Each measurement was performed in duplicate. The thrombin generation curve for the experimental samples was calculated by averaging the duplicates, subtracting the background, converting the fluorescence signal into the AMC concentration and differentiation of the AMC kinetic curve. Consumption of the fluorogenic substrate and the contribution of the α2-macroglobulin were also taken into account.

### Thromboelastography

The assay was performed using a TEG 5000 Hemostasis Analyzer System (Haemoscope; Braintree, MA). The effects of the Mutant B dose were determined on frozen-thawed samples of normal PPP and reconstituted samples of lyophilized fXII-depleted plasma (Renam). To study contact-activated clotting, 330 μl of normal PPP was pre-incubated at 37°C with various concentrations of Mutant B, CTI, or vehicle and placed in a warmed cup containing 30 μl of 200 mM CaCl_2_. In the experiments studying TF-dependent coagulation, 300 μl of fXII-depleted plasma pre-incubated with fXIIa inhibitors was placed in a cup containing both 30 μl of 200 mM CaCl_2_ and 30 μl of TF (Renam; final plasma concentration 0.6 pM in a total volume of 360 μl). The measurement of the clot viscoelasticity lasted for 80 min. The values for reaction time R (min), clot formation time K (min), the α angle (°), and the maximum amplitude MA (mm) were analyzed.

### Thrombodynamics assay

The impact of the fXIIa inhibitors on the spatial dynamics of clot growth was studied using a Thrombodynamics Analyzer; the device and the assay reagents were from HemaCore (Moscow, Russia). The assay was performed as previously described in detail [34,36,37]. Briefly, 120 μl of frozen-thawed PFP or reconstituted lyophilized plasma sample was mixed with the fXIIa inhibitor (Mutant B, CTI) or a vehicle (13.3 μl) and incubated at 37°C for 15 min. Plasma was placed in a tube containing lyophilic calcium acetate (final concentration in citrated plasma 20 mM that corresponded to a concentration of free Ca^2+^ ions 2 mM). After re-calcification, this mixture was placed into a flat transparent assay cuvette and the assay was started immediately since the plasma was brought in contact with an activator coated with surface-immobilized TF [38]. Clotting in the assay cuvette filled with plasma was monitored for 30 min using dark-field video microscopy. A series of the cuvette images that visualized the growing fibrin clots were processed. For a clot grown from the immobilized TF (“TF-initiated clot”), the size in the direction of growth (μm) versus time (min) was plotted, while for the TF-independent clots grown far from the activator, the cuvette area occupied by the clots (%) was plotted versus time (min).

### Statistical analysis

Data were analyzed with OriginPro software (Microcal Software; Northampton, MA). The data presented are the mean of at least 3 repeats ± standard deviation (SD), unless otherwise noted. Prolongation of the whole blood clotting time by fXIIa inhibitors was analyzed by the paired-sample Wilcoxon signed rank test.

## Results

### Selectivity of the fXIIa inhibitors

**Fig 1:**
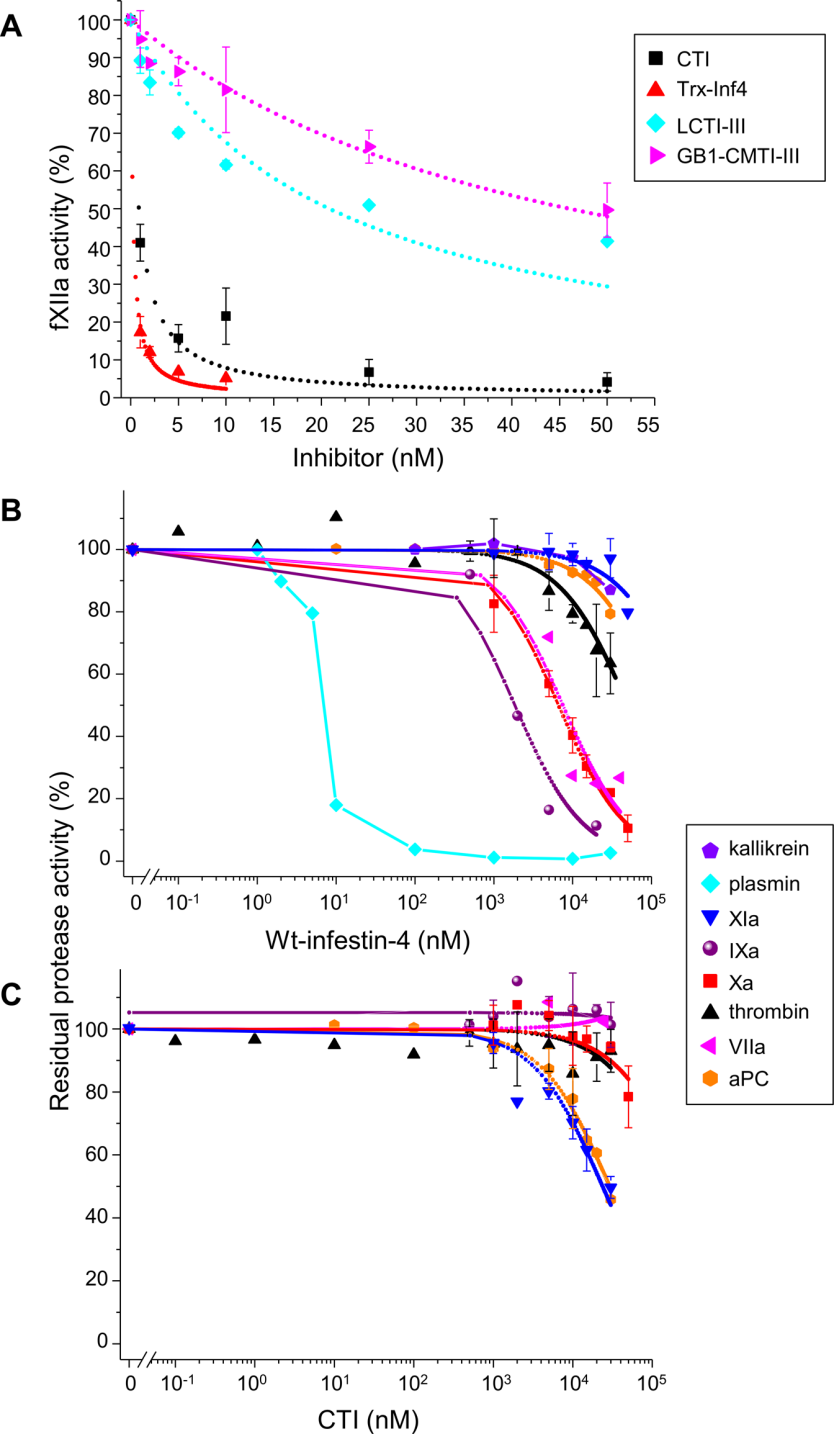
Wt-Inf4 and CTI are the most potent inhibitors of fXIIa and have some off-target activities. **(A)** The residual amidolytic activity (%) of fXIIa upon incubation with various concentrations of wt-Inf4 (red), CTI (black), LCTI-III (cyan), or CMTI-III (magenta). **(B, C)** The residual amidolytic activity (%) of the coagulation-related proteases plasma kallikrein (purple pentagon), plasmin (cyan), fXIa (blue), fIXa (purple sphere), fXa (red), thrombin (black), fVIIa (magenta), and aPC (orange) upon incubation with various concentrations (logarithmic scale) of wt-Inf4 **(B)** and CTI **(C)**.

First, we estimated the degree of selectivity of the wild type infestin-4 against serine proteases of blood coagulation. The wt-Inf4 inhibitory activity against purified fXIIa was measured in the chromogenic assay and compared with other potent inhibitors of fXIIa: corn trypsin inhibitor (CTI) from the cereal family and the squash-family trypsin inhibitors from *C*. *maxima* (CMTI-III) and *L*. *cylindrica* (LCTI-III) (Fig 1A). Wt-Inf4 strongly inhibited fXIIa with an inhibitory constant *K*_*i*_ = 0.113 ± 0.023 nM, which agreed with previously published values [13,15]. CTI inhibited fXIIa approximately 4-fold less, while both CMTI-III and LCTI-III had *K*_*i*_ values of 10 nM or greater. The off-target activity of wt-Inf4 and CTI were measured in the chromogenic assay with a coagulation factor (XIa, IXa, Xa, thrombin, or VIIa) or a coagulation-related protease (plasma kallikrein, plasmin, tPA, or aPC). Wt-Inf4 was a strong inhibitor of plasmin (*K*_*i*_ 4.7 ± 1.5 nM) and a weak inhibitor of fXa (2.43 ± 0.87 μM), fIXa (1.06 ± 0.25 μM), fVIIa (4.7 ± 1.7 μM), and thrombin (33.4 ± 9.4 μM) (Fig 1B), but CTI weakly inhibited fXIa (15.4 ± 2.6 μM) and aPC (15.9 ± 3.8 μM) (Fig 1C). Furthermore, proteins tested inhibited neither plasma kallikrein nor tPA up to a concentration of 30 μM. CTI had approximately 40,000-fold selectivity, and wt-Inf4 had approximately 10,000-fold selectivity against human coagulation factors (if to ignore its inhibition of plasmin).

### Design of infestin-4 mutants

**Fig 2:**
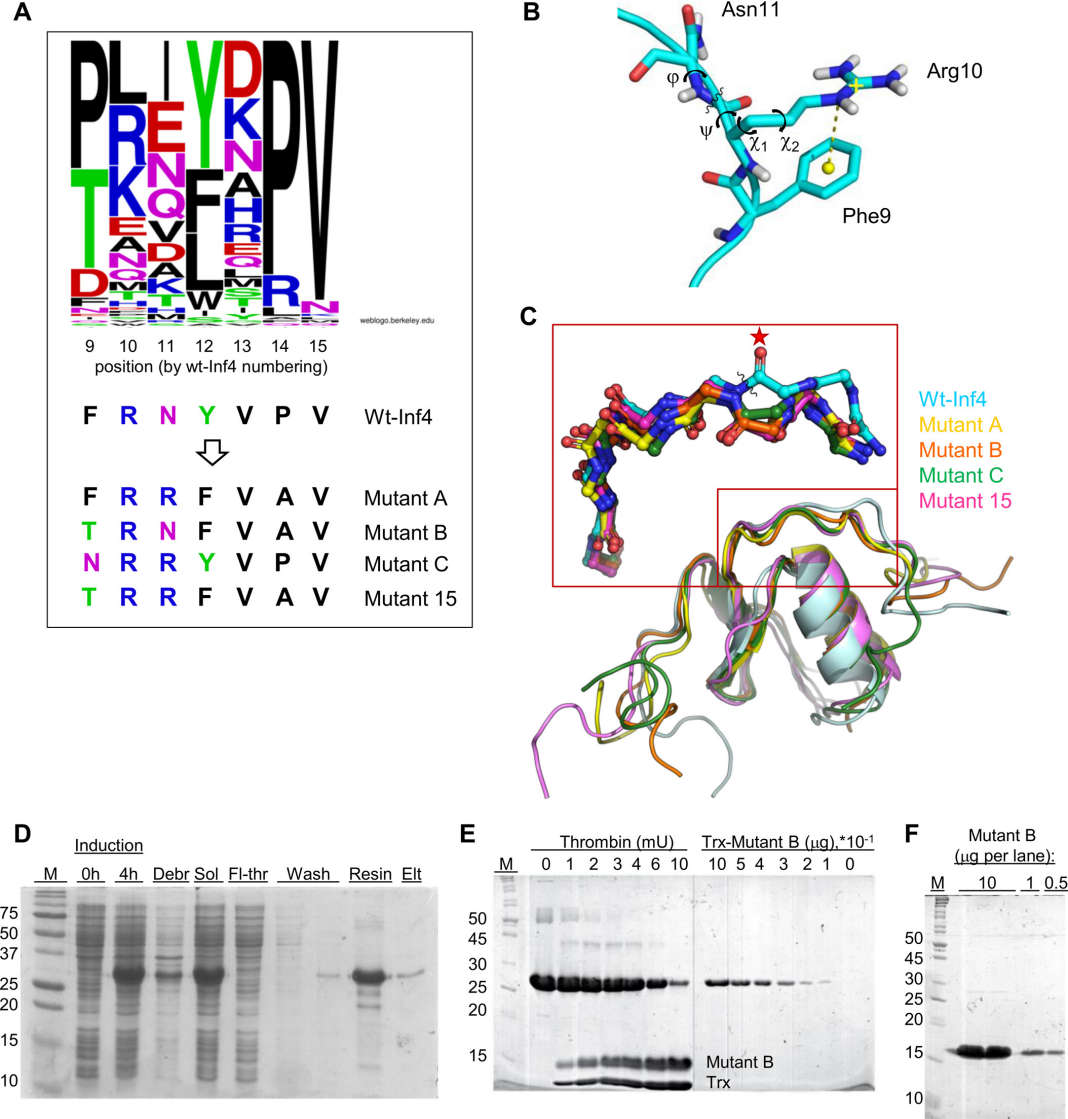
Design of mutations in the reactive site region of Inf4. **(A)** A web-logo representation [41] of the P^2^–P^5’^ binding loop sequences of 89 proteinase inhibitors from the Kazal family [39]. The amino acid positions are numbered by the wt-Inf4 sequence Phe9–Val15. Sequences of the corresponding reactive site fragments from wt-Inf4 and its mutants are shown below. **(B)** The MD simulation indicated a π-cation interaction (yellow dashed line) in wt-Inf4 between the phenyl group of Phe9 and the guanidinium group of Arg10. The nitrogen atoms are shown in blue, oxygen in red, and hydrogen in white. The torsion angles ψ, ϕ, χ_1_, and χ_2_ that describe the scissile bond conformation and rotations of the Arg10 side chain are drawn. **(C**) Superposition (main chain atoms) of wt-Inf4 (the most abundant conformation over the MD trajectory) with the structures of Inf4 mutants whose reactive loop solely adopted the canonical conformation. A close-up view of the reactive site region is indicated with a rectangle (wt-Inf4 –cyan, Mutant A–yellow, Mutant B–orange, Mutant C–green, Mutant 15 –magenta; this coloring is also used in the following Figs). The scissile bond of wt-Inf4 is indicated with a wavy line; a star indicates the Arg10 carbonyl oxygen. **(D)** A Coomassie-stained 12.5% SDS-PAGE showing the one-step purification procedure for one of the Inf4 versions expressed in *E*. *coli* BL21(DE3) cells (Trx-Mutant B is shown as an example). The protein samples applied to the gel are as follows: M–molecular weight marker (kDa); Induction 0 h and 4 h–cell lysates before 1 mM IPTG induction and 4 h later, respectively; Debr and Sol–insoluble and soluble fractions, respectively; Fl-thr–flow-through of the Sol fraction applied to the column; Wash–washing the column with 50 mM Tris, pH 7.5, 500 mM NaCl, 100 mM imidazole; Resin–Chelating Sepharose with bound and washed Trx-Mutant B (26 kDa); Elt–Chelating Sepharose after Trx-Mutant B elution with 50 mM Tris-HCl, pH 7.5, 500 mM imidazole; thereafter, the protein sample was applied on the SOURCE 30Q column. **(E)** Following the two-step purification, the Trx-fused protein was cleaved with bovine thrombin. Thrombin (mU)–indicated amounts of bovine thrombin (0, 1, 2, 3, 4, 6, and 10 milliunits) were added to 10 μg of Trx-Mutant B resulting in two separate bands of Mutant B (13.1 kDa) and Trx (12.9 kDa). **(F)** Mutant B–indicated quantities of the Mutant B protein (10, 1, and 0.5 μg loaded into gel) that was purified after the cleavage reaction, as described in the sub-section “Expression of the thioredoxin-fused infestin-4 and its mutants”.

Prior to testing infestin-4 as a reagent to block *in vitro* contact activation, we attempted to eliminate its off-target activity by introducing mutations in its reactive site region. First, to design the mutants, we analyzed the individual amino acids in the reactive site sequences of 89 inhibitors from the Kazal family [39], including infestin-4 (P^2^ –P^4’^ sequence FRNYVP). The comparison revealed that the Phe9 residue found at the P^2^ reactive site position of wt-Inf4, very rarely appeared at this position in the Kazal-type inhibitors (Fig 2A). In addition to wt-Inf4, Phe was found at the P^2^ position in only the sequences of dipetalogastin_6 and brasiliensin_8, which are predicted domains with unknown activities. The P^2^ position was more frequently occupied by either Pro or Thr; the latter is known to form hydrogen bonds with the P^1’^ residue thus promoting the canonical conformation of an inhibitor [40]. Moreover, P^2^ Thr was contained in an infestin-4 Mutant 15 (P^2^ –P^4’^ sequence TRRFVA), which was previously reported [16]. To elucidate whether the uncommon P^2^ Phe9 residue affected the structure of Inf4, we applied a 150 ns molecular dynamics (MD) simulation to the crystal structure of wt-Inf4 (PDB ID 2ERW). The simulation revealed that the side chains of the Phe9 and Arg10 residues appeared in close proximity to each other; the distance between these groups (4 Å) is shown as a yellow dashed line in Fig 2B and indicates a π-cation interaction between the Phe9 and Arg10 residues. It was unusual that there was no indication of the typical interaction between the P^2^ and P^1’^ residues in wt-Inf4; this interaction occurs in a majority of the canonical inhibitors [40]. Hence, we designed mutants of Inf4 where Phe9 was substituted by a more common Thr residue (Mutant B) or by Asn, another hydrogen forming residue (Mutant C). In Mutant B, Asn11 (P^1’^ position) was conserved, while Asn11 was substituted by Arg in Mutant C because Arg11 was present in a majority of fXIIa-binding peptides from the study [16], including Mutant 15. Phe9 was conserved in Mutant A, whereas Asn11 was substituted by Arg in this mutant. Additionally, the P^2’^ Tyr and P^4’^ Pro residues of wt-Inf4 were substituted by Phe and Ala residues, respectively, in Mutant A and Mutant B (for a full list of the P^2^ –P^5’^ sequences see Fig 2A). Arg10 at the P^1^ position, known to determine the general specificity for the trypsin-like proteases, was preserved in all Inf4 variants.

### Expression of thioredoxin-fused infestin-4 and its mutants

The mutants were analyzed using the MD simulation, and it showed that in Mutant B, ‘canonical’ hydrogen bonds were formed between the P^2^ Thr9 and P^1’^ Asn11 residues (the distance between the Thr9 side chain oxygen and the P^1’^ Asn11 main chain nitrogen was mostly in a range of 3–5 Å over the MD trajectory). Similar interactions between the P^2^ and P^1’^ residues were proposed in Mutant C and Mutant 15. Even in Mutant A, where Phe9 was conserved, this residue interacted with the P^1’^ Arg11 (the distance between their side groups approached 4–5 Å) than with P^1^ Arg10 (distance was ≈8 Å). Then, structures of the Inf4 mutants overlapped with the model structure of wt-Inf4 (Fig 2C). The scissile bond region of wt-Inf4 (labeled with a wavy line) differed significantly from the canonical conformations of the Inf4 mutants, as well as from the other inhibitors CTI (the MD trajectories were from [10]), CMTI-III, and LCTI-III. As shown in Fig 2C, the Arg10 carbonyl oxygen of wt-Inf4 (labeled with a star) was oriented in the opposite direction compared to the other structures.

After the analysis was performed, the Inf4 variants were expressed in *E*. *coli* BL21 (DE3) cells from a pET32a vector as thioredoxin (Trx)-fused proteins. These recombinant proteins were purified using nickel-chelating chromatography (Fig 2D). Some additional bands were present in those preparations, and an ion-exchange chromatography step was used to further purify the fusion-proteins to homogeneity (Fig 2E; right half). The approximate yield of the homogeneous proteins was 20–60 mg per liter of cell culture. In some coagulation experiments, Mutant B was cleaved from the Trx-fusion with thrombin (Fig 2E; left half) and purified to homogeneity (Fig 2F).

### Inhibition of fXIIa by the mutants

**Fig 3:**
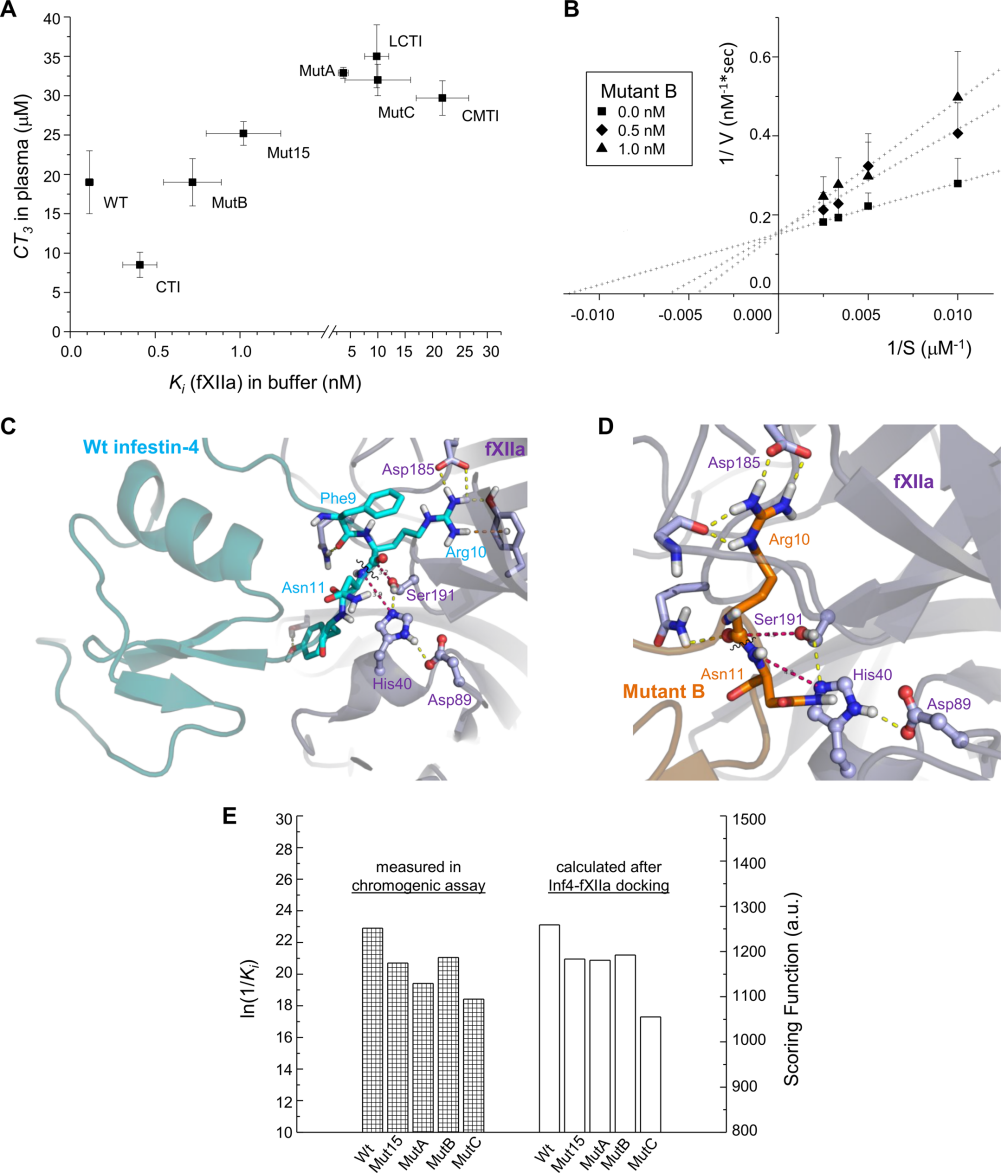
Analysis of fXIIa binding by Inf4 variants. **(A**) The *CT*_*3*_ values (μM) measured in the aPTT assay in normal plasma are plotted against the *K*_*i*_ values for the Inf4 variants and the inhibitors of fXIIa from other families (CTI, CMTI-III, LCTI-III). The mean values ± SD are presented (n = 3). (**B**) Lineweaver-Burk plot (1/V, nM^-1^*sec, versus 1/S, μM^-1^) of fXIIa inhibition by 0, 0.5 and 1 nM of Mutant B at various concentrations (100, 200, 300, and 400 μM) of S-2302 substrate (mean + SD values, n ≥ 3). The data fitting with linear functions is shown with dots. (**C, D)** Complex of fXIIa (light violet) docked with wt-Inf4 (**C**) and Mutant B (**D**). The P^1^ Arg10 residue of these inhibitors formed a salt bridge with S^1^ Asp185 of fXIIa and the scissile bond was close (3.2–3.9 Å) to the catalytic residues Ser191 and His40. **(E)** A diagram representing the mean values of ln(1/*K*_*i*_) (left axis) (n = 3; SD was approx. 0.2, i.e., 1%), where *K*_*i*_ is the inhibitory constant of the Inf4 variants for fXIIa; this value is proportional to the energy term. The values of the energy-like scoring function *SF* (right axis) were calculated with the ClusPro server.

We characterized the inhibitory activities of the expressed mutants. To assess the inhibitory activity of recombinant Inf4 mutants against fXIIa, they were incubated with fXIIa in a buffered solution, followed by the addition of the S-2302 chromogenic substrate (Table 1) and measurement of the residual amidolytic activity of fXIIa. To address the ability of the Inf4 mutants to inhibit contact-activated clotting in blood plasma, they were incubated with citrated pooled plasma followed by 3-min incubation with kaolin and phospholipids, re-calcification, and measurement of the activated partial thromboplastin time (aPTT). The clotting time was dose-dependently prolonged by the inhibitors, and the *CT*_*3*_ value was defined as the concentration of inhibitor that prolonged the clotting time by 3-fold compared to the clotting time in plasma without inhibitor (47 s in normal plasma). The *CT*_*3*_ value was drawn on the plot for each inhibitor versus its *K*_*i*_ value measured in saline (Fig 3A). Mutant B was the most active of the mutants–its *CT*_*3*_ concentration was 19 ± 3 μM, similar to wt-Inf4; nevertheless, its *K*_*i*_ value for fXIIa (0.72 ± 0.17 nM) was 6 times greater than that of wt-Inf4. S1 Fig shows the concentration-dependent inhibition of fXIIa amidolytic activity by Trx-fused Mutant B that was purified by two-step chromatography, as described in Materials and Methods. The Lineweaver-Burk plot (Fig 3B) was created by plotting the inverse rate of S-2302 cleavage by fXIIa (1/V, nM^-1^*sec) versus the inverse concentration of S-2302 (1/S, μM^-1^). This plot yielded three lines at Mutant B concentrations of 0 nM, 0.5 nM, and 1 nM; these lines had the same Y-intercept. It indicated that Mutant B had a competitive mode of action against fXIIa, which was previously reported for wt-Inf4 [15] and CTI [17]. Other Inf4 mutants had the *CT*_*3*_ concentrations between 25 and 32 μM and *K*_*i*_ values in the range 1–10 nM (Fig 3A). For comparison, CTI had a *CT*_*3*_ of 8.5 ± 1.6 μM and a *K*_*i*_ towards fXIIa of 0.41 ± 0.10 nM. The corresponding values for the squash-type inhibitors CMTI-III and LCTI-III were also plotted. Moreover, none of the inhibitors prolonged the prothrombin time.

We also analyzed the binding of the Inf4 variants to fXIIa using flexible protein-protein docking; the homology model of fXIIa had been previously constructed [10]. In the complex of wt-Inf4 (cyan) and fXIIa (violet), the P^1^ Arg10 side chain protruded and formed a salt bridge with the Asp185 of the S1 specificity pocket of fXIIa (Fig 3C). The catalytic atoms Ser191 O^γ^ and His40 N^ε2^, which are involved in a nucleophilic attack on the scissile bond, were placed 3.2 and 3.9 Å from C and N atoms of the scissile bond, respectively (red dashed lines). The protease-binding loop was correctly positioned in the catalytic pocket in relation to the triad Ser191, His40, and Asp89; this position corresponds to a strong inhibition of fXIIa by wt-Inf4. Similar correct positions of the protease-binding loops were obtained in the complexes of fXIIa with Mutant B (Fig 3D) and other Inf4 mutants. Values of the scoring function (*SF*), which is dependent on the association energy, were calculated for the docked complexes (Table 2). The *SF* values were correlated with the experimentally measured *K*_*i*_ values for fXIIa (Fig 3E), which may confirm our biochemical data.

**Table 2:** Association of the Inf4 variants with FXIIa. Characterization of the anti-fXIIa activity of wt-Inf4, its mutants and the canonical inhibitors CTI, LCTI-III, and CMTI-III. The **reactive site P** ^**2**^ **–P** ^**4’**^ **sequences** are shown; ***K***_***i***_ values (nM) against fXIIa and ***CT***_***3***_ concentrations (μM) estimated with the aPTT assay are presented as the mean ±SD for n ≥ 3. Dimensionless values of the scoring function ***SF*** are provided as a result of flexible protein-protein docking of these inhibitors with the model fXIIa structure by ClusPro service.

Inhibitor	P^2^ –P^4’^ reactive site	*K*_*i*_ fXIIa (nM)	*CT*_*3*_ (μM)	*SF*
wt-Inf4	FRNYVP	0.113 ±0.023^a^	19 ±4	-1258.9
Mutant A	FRRFVA	3.7 ±0.9	32.9 ±0.7	-1180.5
Mutant B	TRNFVA	0.72 ±0.17	19 ±3	-1192.1
Mutant C	NRRYVP	10 ±6	32 ±2	-1055.1
Mutant 15	TRRFVA	1.02 ±0.22^b^	25.2 ±1.5	-1183.3
CTI	PRLPWP	0.41 ±0.10	8.5 ±1.6	-1266.7
LCTI-III	PRILME	9.8 ±2.2	35 ±4	-1000.2
CMTI-III	PRILMK	21.8 ±4.8	29.7 ±2.2	-971.7

^a^–corresponds to the value published in [16]

^b^–does not correspond to the value published in [16].

### Off-target activities of the Inf4 mutants

**Fig 4:**
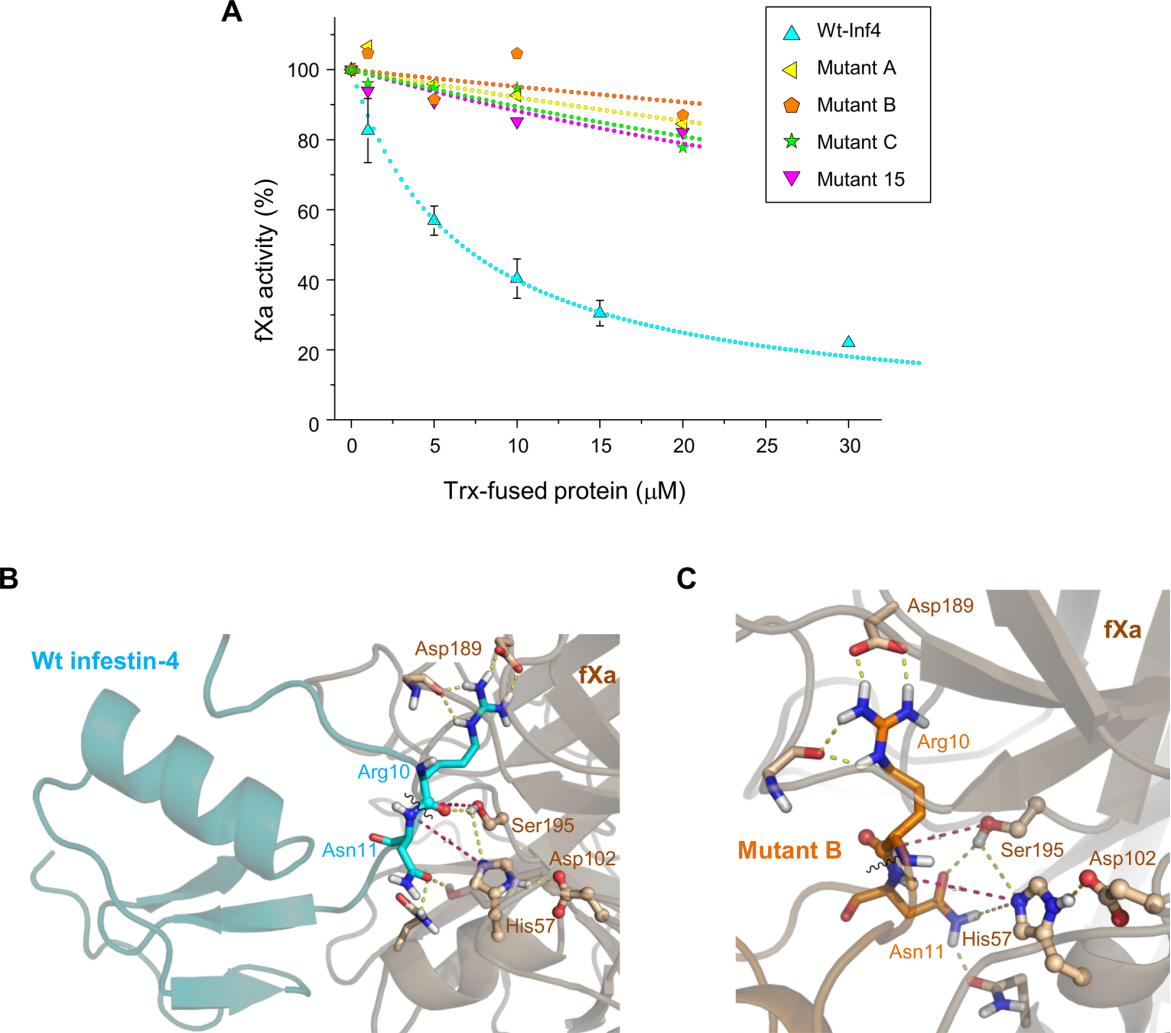
Inf4 mutants did not inhibit FXa. **(A)** Residual amidolytic activity of fXa at various concentrations of the Inf4 variants – 100% activity of fXa corresponds to a *k*_*cat*_ value of 240 s^-1^. The mean ± SD values are shown (n = 3). Data fitting with a hyperbola is shown with dots. **(B, C)** Complexes of either wt-Inf4 **(B**; cyan) or Mutant B **(C**; orange) docked with fXa (secondary structure ribbons and C atoms are beige). Hydrogen bonds and salt bridges between charged functional groups are shown as yellow dashed lines. Red dashed lines show distances between the atoms involved in the cleavage reaction: 1) direction of the nucleophilic attack of Ser195 O^γ^ on the Arg10 carbonyl carbon and 2) direction of the proton transfer from His57 N^ε2^ to the Asn11 carbonyl nitrogen (during the formation of the tetrahedral intermediate, a proton is acquired by His57 from Ser195, then a proton is transferred to the scissile bond to effect the cleavage). The scissile bond (shown as wavy line) of wt-Inf4 **(B)** was located in the fXa pocket properly for formation of the intermediate. P^1^ Arg10 was bound to S^1^ Asp189 and Gly218 by salt bridges. In addition, the Asn11 side chain of Mutant B **(C)** interfered with the catalytic Ser195 O^γ^ and His57 N^ε2^ atoms.

We estimated the impact of the mutations on the off-target activities of infestin-4. In contrast to wt-Inf4, none of the Inf4 mutants inhibited fXa (Fig 4A). Additionally, the Inf4 mutants had only minor, if any, activity against plasmin: their *K*_*i*_ values were 3–4 orders of magnitude greater than that of wt-Inf4 (4.7 ± 1.5 nM). None of the tested versions of Inf4 inhibited fXIa, tPA, plasma kallikrein, and aPC. Mutant A and Mutant 15 did not inhibit thrombin at a concentration up to 30 μM (Table 3), while Mutant B was more active against thrombin (*K*_*i*_ 2.9 ± 1.2 μM) than wt-Inf4 (*K*_*i*_ 33.4 ± 9.4 μM). Nevertheless, compared to wt-Inf4 and the other mutants, Mutant B had the least off-target activity against fIXa and fVIIa (Tables 3 and 4). In general, anti-fXa activity was fully eliminated by mutation of the reactive site of Inf4. Moreover, off-target activities against fIXa, fVIIa, thrombin, and plasmin were significantly reduced in (a subset of) these mutants.

**Table 3:** *K*_*i*_ (μM) values of Inf4 versions against fXa, thrombin, plasmin, and fIXa. ***K***_***i***_ values (μM) are presented as the mean ±SD for n ≥ 3. N.I.–not inhibited at concentrations of up to 30 μM of the inhibitor.

Inhibitor	fXa	thrombin	Plasmin	fIXa
wt-Inf4	2.43 μ0.87 ^d^	33.4 ±9.4 ^d^	0.0047 ±0.0015 ^c^	1.06 ±0.25 ^d^
Mutant A	N.I.	N.I.	17.5 ±4.5	0.32 ±0.14
Mutant B	N.I.	2.9 ±1.2	7.0 ±3.0	4.6 ±1.5
Mutant C	N.I.	28.5 ±8.0	2.45 ±0.6	0.66 ±0.18
Mutant 15	N.I. ^a^	N.I.	28 ±8 ^b^	1.33 ±0.44

^a^–corresponds to the value published in [16]

^b^–does not correspond to the value published in [16]

^c^–corresponds to the value published in [15]

^d^–does not correspond to the value published in [15].

**Table 4:** *K*_*i*_ (μM) values of wt-Inf4, Mutant B, Mutant 15, and CTI against coagulation-related serine proteases. ***K***_***i***_ values (μM) of wt-Inf4, Mutant B, Mutant 15, and CTI against the serine proteases: fXIa, fVIIa, aPC, plasma kallikrein, and tPA, are shown as the mean ±SD for n ≥ 3.

Inhibitor	fXIa	fVIIa	aPC	kallikrein	tPA
wt-Inf4	N.I. ^c^	4.7 ±1.7 ^d^	N.I. ^c^	N.I. ^c^	N.I.
Mutant B	N.I.	N.I.	N.I.	N.I.	N.I.
Mutant 15	N.I.	1.9 ±0.7	N.I.	N.I.	N.I.
CTI	15.4 ±2.6	N.I.	15.9 ±3.8	N.I.	N.I.

^c^–corresponds to the value published in [15]

^d^–does not correspond to the value published in [15].

We attempted to understand why mutants of Inf4 did not inhibit fXa (Fig 4A). Complexes of fXa with Inf4 variants were studied using flexible protein-protein docking. In the complex of wt-Inf4 (cyan) with fXa catalytic domain (beige; PDB ID 2JKH), the P^1^ Arg10 residue formed a salt bridge with the Asp189 residue of the S1 specificity pocket (Fig 4B). The scissile bond of wt-Inf4 was adjacent to the catalytic triad Ser195, His57 and Asp102, 4.4–5.8 Å apart from the Ser195 O^γ^ and His57 N^ε2^ atoms. In the complex of Mutant B (orange) and fXa (beige), where the P^1^ Arg10 was also bound to S^1^ Asp189, the Asn11 residue was able to form hydrogen bonds with the catalytic Ser195 and His57 residues (Fig 4C). We proposed that the hydrogen bonding of Asn11 with the catalytic residues might prevent the nucleophilic attack on the scissile bond, thus interfering with the standard mechanism of inhibition [42]. Such interference could result in a weak noncovalent binding of Mutant B with fXa and a loss of the inhibitory activity. Similar ‘interfering’ interactions with the catalytic residues of fXa were also proposed for the remaining Inf4 mutants (data not shown).

### Specific repression of contact-activated clotting in blood and plasma by Mutant B

**Fig 5:**
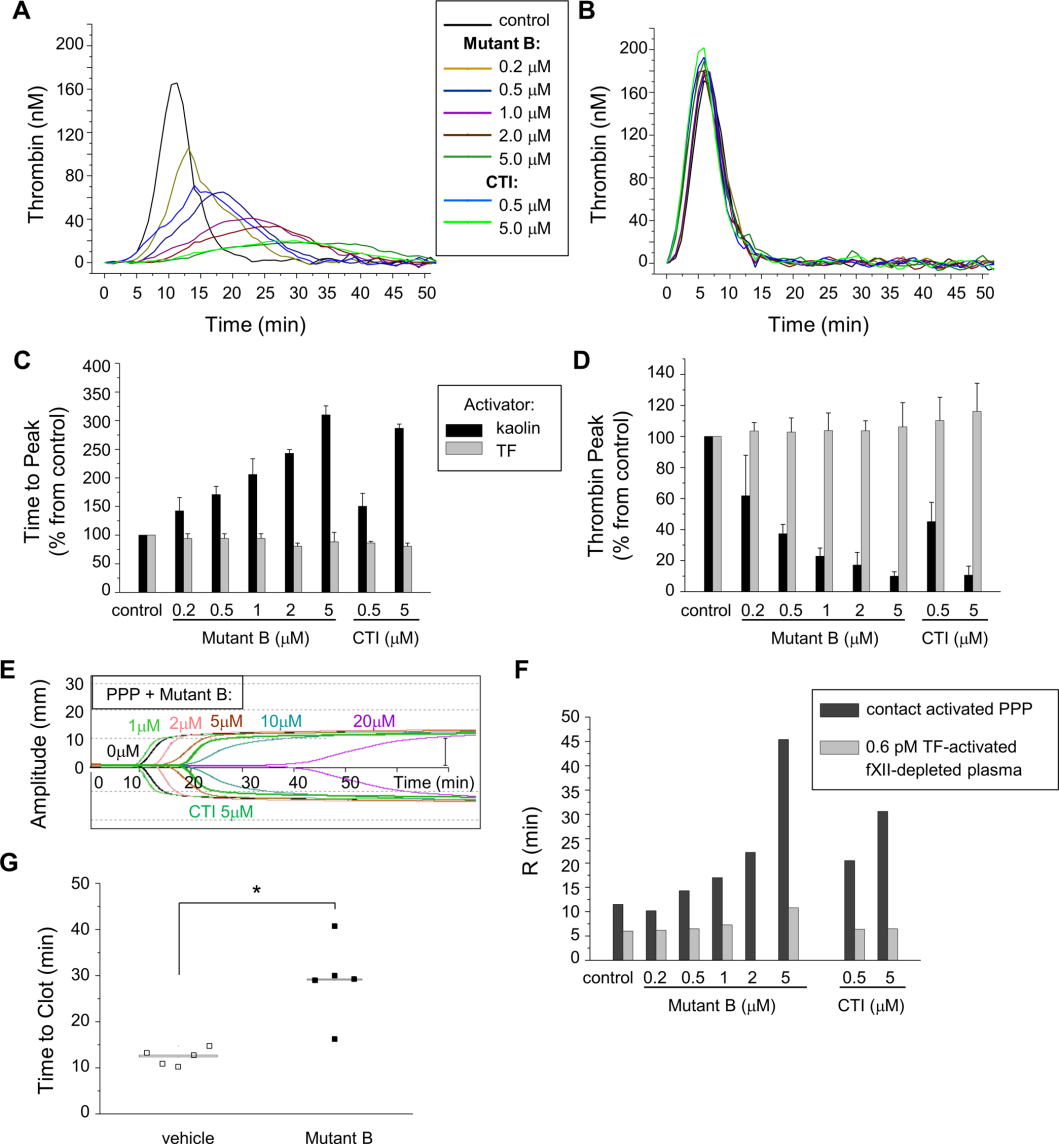
Mutant B selectively inhibits the contact pathway of coagulation. **(A–D**) Thrombin generation in normal platelet-poor plasma was triggered with a mixture of calcium chloride and either kaolin (**A**) or TF (5 pM final concentration) **(B)** and was monitored for 90 min. Frozen-thawed plasma was preincubated at 37°C with various concentrations of Mutant B as indicated in the legend (0.2, 0.5, 1, 2, and 5 μM), CTI (0.5 and 5 μM) or vehicle (control). The assay parameters including the time to the thrombin peak **(C)** and the peak amplitude **(D)** were calculated for the experiments with the activation by kaolin (black bars) or 5 pM TF (gray bars). The mean values ± SD are shown; each experiment was performed in duplicate and repeated twice. **(E, F)** The thromboelastography assay was carried out in plasma preincubated with Mutant B (1, 2, 5, 10, and 20 μM), CTI (5 and 15 μM), or vehicle (control). **(E)** Thromboelastogram representing clotting in frozen-thawed platelet-poor plasma from healthy volunteers, drawn into a flask without any activator (coagulation was triggered by the contact pathway from flask walls). **(F)** Diagram showing the mean R-time values (n = 2) for thromboelastograms, obtained as a result of the assay either in normal platelet-poor plasma without activator (black bars), or in fXII-depleted plasma activated with 0.6 pM TF (gray bars). **(G)** Prolongation of the whole blood clotting time by Mutant B. The time to the visually detected clotting was measured in whole blood collected into tubes, which were prefilled with either vehicle (final concentration in blood 30 mM Hepes pH 7.4, empty squares) or Mutant B (10 μM, filled squares), without any other anticoagulant. Individual data from 5 donors and the median values are presented; for each donor the experiment was performed in duplicate. P-value (* < 0.005) was estimated using the Wilcoxon rank test.

Finally, we addressed the potential value of Mutant B, one of the most potent Inf4 mutants, as a new anticoagulant. Using a set of the global assays, we investigated whether this mutant, which weakly inhibited thrombin in saline, could be used in plasma to repress the contact pathway without affecting TF-dependent coagulation (i.e., the extrinsic pathway). For better reliability, Mutant B was cleaved and purified from Trx for these experiments (Fig 2E and 2F). The thrombin generation assay triggered by either kaolin, an activator of the contact pathway (Fig 5A), or 5 pM TF (Fig 5B) revealed that Mutant B did not affect TF-dependent generation in normal plasma, whereas it repressed the contact-dependent generation of thrombin. According to the competitive mode of action, Mutant B dose-dependently prolonged the time to reach the thrombin peak (Fig 5C) and reduced the thrombin amplitude (Fig 5D) when coagulation was activated by kaolin. The effective concentration of Mutant B was approx. 5 μM, similar to that of CTI. Similar effects were observed in the thromboelastography assay. Mutant B as well as CTI, dose-dependently delayed clotting in normal plasma triggered by contact with the assay cuvette (Fig 5E). These inhibitors dose-dependently prolonged the ‘R-time’ value in normal plasma without the addition of an activator (Fig 5F, dark bars) while both inhibitors had no effect on clotting in fXII-depleted plasma triggered by 0.6 pM TF (Fig 5F, light bars). In another experiment, when blood was collected into plastic tubes pre-filled with either Mutant B or the vehicle, whole blood clotting time was visually measured. Clots in the whole blood might appear due to the contact with tube walls. Clotting became visible at 29.1 ± 8.9 min after the collection of blood into tubes containing Mutant B (similarly to the effect of CTI; data not shown) compared to at 12.4 ± 1.8 min for tubes without an inhibitor (Fig 5G). In this experiment, the calculated concentration of Mutant B in plasma was 20 μM (near its *CT*_*3*_ value), and no other anticoagulant (like sodium citrate) was used.

**Fig 6:**
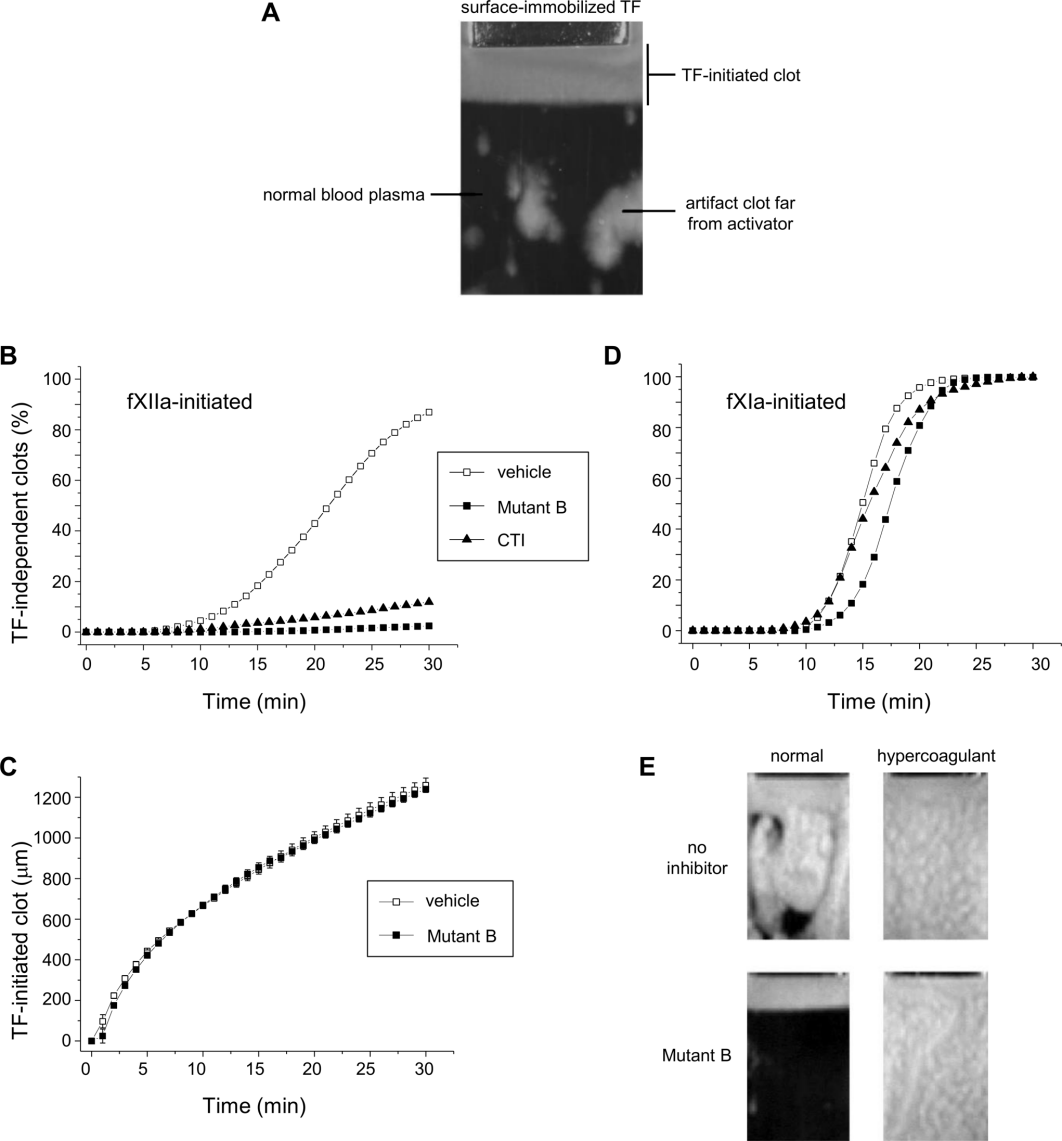
Application of Mutant B in the thrombodynamics assay. The thrombodynamics assay in platelet free plasma. **(A)** A representative dark-pole image of the assay cuvette filled with recalcified normal plasma (dark background) 30 min after the start of the thrombodynamics assay. TF was localized on the surface of a rectangular insert at the upper side of the cuvette (“Surface-immobilized TF”) and triggered a frontal growth of the bright fibrin clot (“TF-initiated clot”) in the downwards direction. Additional TF-independent artifact clots appeared far from the activator. **(B)** A percentage of the cuvette area occupied by fXIIa-initiated, TF-independent artifact clots in frozen-thawed normal plasma versus time (min). If no fXIIa inhibitor was added, then these clots appeared (occupied 5% of the cuvette area) 10 min after the assay was initiated and occupied the entire cuvette (100% of the cuvette area) 30 min later. Plasma samples were pre-incubated at 37°C with a vehicle (empty squares) or Mutant B (filled squares) and CTI (filled triangles) at their *CT*_*3*_ concentrations: 20 μM and 10 μM, respectively. The mean values are presented for n = 10. **(C)** The size of the TF-initiated fibrin clot in a downward direction (μm) versus time (min) in fXII-depleted plasma. The plasma was preincubated at 37°C with Mutant B (20 μM, filled triangles) or vehicle (empty squares; mean values for n = 3 are shown). **(D)** A percentage of the cuvette area occupied by fXIa-initiated, TF-independent clots in frozen-thawed normal plasma versus time (min). These clots appeared approximately 10–12 min after the assay was initiated and occupied the entire cuvette 20 min later. The plasma samples were preincubated with a vehicle, Mutant B (20 μM) or CTI (10 μM). The mean values are presented for n = 10. **(E)** Images of the assay cuvettes 30 min after the assay was initiated, filled with normal frozen-thawed plasma that was spiked with 20 pM fXIa (“hyper”) or vehicle (“normal”) and preincubated with Mutant B (“MutB”) or vehicle (“no inhibitor“).

The use of Mutant B as a reagent for *in vitro* assays was also demonstrated with the thrombodynamics assay. In this assay, re-calcified plasma was placed in the assay cuvette, where a frontal growth of fibrin clot occurred from the surface with immobilized TF (Fig 6A). After re-calcification when no inhibitor of contact activation was applied, the problem was fXIIa autoactivation, which, as we proposed, occurred on walls of the assay cuvette and represented an artifact. It resulted in generation of TF-independent clots, which appeared in a bulk of normal plasma far from the TF localization site. We tested whether the inhibition of fXIIa by Mutant B would repress these fXIIa-initiated, artificial clots. The addition of either 20 μM Mutant B or 10 μM CTI into normal plasma successfully prevented the appearance of these contact-activated clots during a 30 min experimental time (Fig 6B). Moreover, Mutant B did not affect the growth of the TF-initiated clot both in normal and fXII-depleted plasma samples (Fig 6C) (as well as in fVIII- and fIX-deficient plasma; data not shown). However, in a patient’s plasma some TF-independent clots were formed even in the presence of a sufficient amount of the fXIIa inhibitor [37]. It was proposed that in this case, TF-independent clots were initiated by both fXIIa (generated during plasma handling) and circulating fXIa; the latter could be a marker of a hypercoagulant state [35]. Therefore, we used 20 pM fXIa added into normal plasma as a model of hypercoagulation. Neither Mutant B nor CTI prevented the appearance of the fXIa-initiated, TF-independent clots in this modeled plasma (Fig 6D). To visualize these experiments, we present images of the assay cuvette in 30 min after assay start in Fig 6E. If no inhibitor was added (upper panel), TF-independent clotting in normal plasma (initiated by autoactivation of fXIIa) was poorly distinguished from the clotting initiated by fXIa. In contrast, when plasma was pre-incubated with Mutant B (20 μM), clotting in ‘hypercoagulant’ plasma could be clearly distinguished from clotting in normal plasma, where no ‘artificial’ fXIIa activation occurred. From our point of view, all of these data provide a body of evidence regarding the sufficient selectivity of Mutant B, an improved variant of infestin-4, in plasma. These results particularly indicate that Mutant B can be used as a novel reagent to repress *in vitro* contact activation.

## Discussion

Factor XIIa, plasma kallikrein, and kininogen constitute the contact system, which has numerous roles in vascular biology, inflammation, fibrinolysis, and coagulation; among others. This system may function as a defense mechanism to protect the host organism from invasion. FXIIa mediates the release of bradykinin (a blood pressure regulating peptide), binds to endothelial cells and stimulates angiogenesis; additionally, it proteolytically activates plasminogen and fXIa and can stimulate the release of elastase from neutrophils and the secretion of interleukins by monocytes [43]. As fXIIa is readily (auto)activated on exogenous surfaces, it is responsible for thrombotic complications following the use of extracorporeal circulation devices [7]. Additionally, contact activation complicates the *in vitro* manipulation of blood and can interfere with TF-triggered clotting during the investigation of coagulation dynamics [8,44]. Therefore, a fXIIa inhibitor would be advantageous in these conditions. In this study, we eliminated (a part of) the off-target activities of infestin-4, a fXIIa inhibitor from a blood sucking insect, to adapt it for the *in vitro* assays. Amino acid substitutions in the reactive site region of infestin-4 were based on their appearance in both the Kazal family [39] and the fXIIa binding peptides as reported by Campos et al. [16]. As analyzed by MD simulation, these mutations (predominantly substitution of Phe9) could lead to the stabilization of the canonical conformation of the reactive site region (Fig 2). These mutations led to a variable decrease in the off-target activities towards fIXa, thrombin, fVIIa (Table 3). Moreover, none of the mutants inhibited fXa or plasmin. Analysis of the binding between the Inf4 variants and fXa using protein-protein docking implied that interference of the side chain groups with the catalytic triad could possibly lead to a loss of this off-target activity in the Inf4 mutants (Fig 4).

Mutant B was the most potent inhibitor of fXIIa of all of the mutants. Additionally, it was a weak inhibitor of thrombin; however, its anti-thrombin activity in saline was greater than that of wt-Inf4. Nevertheless, Mutant B was tested in plasma in various coagulation assays. This inhibitor extensively blocked contact activation: 5 μM of Mutant B delayed plasma clotting in aPTT and thromboelastography assays and delayed thrombin generation initiated by the contact pathway (Fig 5), 10 μM of Mutant B prolonged the storage time of whole blood, and 20 μM of Mutant B prevented formation of contact-activated, artificial clots in normal plasma in the thrombodynamics assay. These effects of Mutant B on the contact-activated coagulation were concentration-dependent (Fig 5C–5F) and can be attributed to the competitive mode of action against fXII (Fig 3B). Hence, we assume that Mutant B blocks the fXIIa activity in a reversible manner; this could be advantageous in some cases when the inhibitor should be removed from plasma prior to an experiment. Moreover, in all these assays, Mutant B did not affect TF-triggered coagulation. In general, these results indicated a substantial selectivity of Mutant B towards fXIIa in plasma. We suggest that Mutant B can become a valuable reagent to simplify operations with blood and improve global coagulation assays [45].

We should also discuss the agreement between the *K*_*i*_ values measured in our study with those reported earlier. *K*_*i*_ values of 0.1 nM and 4.7 nM were obtained in the anti-fXIIa and anti-plasmin inhibitory assays of wt-Inf4, respectively, which agreed with previously published values [13,15]. The *K*_*i*_ value of wt-Inf4 against fXa was equal to 2 μM (Table 3), which is greater than the published values of 0.05 μM [13] or 0.2 μM [15]. This difference could be attributed to the different fusion partners of wt-Inf4 used in these studies. Nevertheless, the lack of the inhibitory activity against fXa and plasmin exhibited by Mutant 15 was in accordance with the study [16]. Although our experiments revealed that anti-fXIIa activity of Mutant 15 was 10-fold less than that of wt-Inf4, which contradicts the reported *K*_*i*_ value of 3.6 pM [16], our *in vitro* data corresponded to the *SF* values obtained *in silico*. Furthermore, consideration of the designed set of mutants that had similar substitutions to Mutant 15 revealed a variable reduction of their anti-fXIIa activity. One possible explanation of this discrepancy is the inaccuracy of the *K*_*i*_ measurement when the inhibitory constant is much lower than the concentration of fXIIa used in the chromogenic assay.

In a previous study [16], only two mutants of Inf4, mutant 15 and mutant 3, were expressed and tested in chromogenic assays against a limited set of proteases: fXIIa, trypsin, fXa, and thrombin. In this study we used the previously reported Mutant 15 as a control. Compared to other studies on infestin-4, the advantages of our study are that a) it evaluated the impact of infestins on plasma coagulation using a set of global coagulation assays, b) it analyzed the structural differences between wt-infestin-4 and its mutants, and c) it analyzed their interactions with the cognate protease (fXIIa) and all other coagulation-related proteases. However, the general question of which mutations should be introduced into the inhibitor loop to eliminate the off-target activity is still unanswered. Determinants of Inf4 selectivity are of particular interest because it is one of the most selective inhibitors of fXIIa and has a potential use as either an anti-thrombotic drug candidate [46] or a component of *in vitro* diagnostic and plasmapheresis systems. Although CTI is currently used in various coagulation assays, we used Inf4 because its selectivity can be adjusted by the mutation of the protease-binding loop and because of the ease of the protein preparation.

## Supporting Information

S1 Fig Inhibition of FXIIa by Mutant B purified with two-step chromatography. Residual amidolytic activity of fXIIa at various concentrations of Trx-fused Mutant B (0 nM; 0.25 nM; 0.5 nM; 1.0 nM; 2.0 nM; 4.0 nM; 8.0 nM). The mean ± SD values are shown (n = 2); data fitting with a hyperbola is shown with dots.
(PDF)

S1 Table Designed primers for cloning the genes of infestin-4, its mutants and CMTI-III. Restriction sites are shown in *italics*; overlapping complementary parts of the primers are underlined; and mutations introduced in the infestin-4 sequence are in **bold**.
(PDF)

## References

[1] Renné T, Schmaier AH, Nickel KF, Blombäck M, Maas C. In vivo roles of factor XII. Blood. 2012;120(22):4296–303. 10.1182/blood-2012-07-292094 22993391 PMC3507141

[2] Schmaier AH. The elusive physiologic role of Factor XII. J Clin Invest. 2008;118(9):3006–9. 10.1172/JCI36617 18725991 PMC2518076

[3] Schousboe I. Pharmacological regulation of factor XII activation may be a new target to control pathological coagulation. Biochem Pharmacol. 2008;75(5):1007–13. 17996217 10.1016/j.bcp.2007.10.003

[4] Renné T, Pozgajová M, Grüner S, Schuh K, Pauer H-U, Burfeind P, et al Defective thrombus formation in mice lacking coagulation factor XII. J Exp Med. 2005;202(2):271–81. 16009717 10.1084/jem.20050664 PMC2213000

[5] Matafonov A, Leung PY, Gailani AE, Grach SL, Puy C, Cheng Q, et al Factor XII inhibition reduces thrombus formation in a primate thrombosis model. Blood. 2014;123(11):1739–46. 10.1182/blood-2013-04-499111 24408325 PMC3954054

[6] Leung PY, Hurst S, Berny-Lang MA, Verbout NG, Gailani D, Tucker EI, et al Inhibition of Factor XII-Mediated Activation of Factor XI Provides Protection Against Experimental Acute Ischemic Stroke in Mice. Transl Stroke Res. 2012;3(3):381–9. 10.1007/s12975-012-0186-5 23634198 PMC3637928

[7] Yau JW, Stafford AR, Liao P, Fredenburgh JC, Roberts R, Brash JL, et al Corn trypsin inhibitor coating attenuates the prothrombotic properties of catheters in vitro and in vivo. Acta Biomater. 2012;8(11):4092–100. 10.1016/j.actbio.2012.07.019 22824529

[8] Luddington R, Baglin T. Clinical measurement of thrombin generation by calibrated automated thrombography requires contact factor inhibition. J Thromb Haemost. 2004;2(11):1954–9. 15550027 10.1111/j.1538-7836.2004.00964.x

[9] Spronk HMH, Dielis AWJH, Panova-Noeva M, van Oerle R, Govers-Riemslag JWP, Hamulyák K, et al Monitoring thrombin generation: Is addition of corn trypsin inhibitor needed? Thromb Haemost. 2009;101(6):1156–62. 19492161

[10] Korneeva VA, Trubetskov MM, Korshunova A V, Lushchekina S V, Kolyadko VN, Sergienko O V, et al Interactions outside the proteinase-binding loop contribute significantly to the inhibition of activated coagulation factor XII by its canonical inhibitor from corn. J Biol Chem. 2014;289(20):14109–20. 10.1074/jbc.M114.553735 24706752 PMC4022879

[11] Kolyadko VN, Korneeva VA, Ataullakhanov FI, Panteleev MA. Molecular mechanisms of thrombosis. Fundamental and applied aspects of the contact activation. Biochem Suppl Ser A Membr Cell Biol. 2014;8(4):279–89.

[12] Campos ITN, Amino R, Sampaio CAM, Auerswald EA, Friedrich T, Lemaire H-G, et al Infestin, a thrombin inhibitor presents in Triatoma infestans midgut, a Chagas’ disease vector: gene cloning, expression and characterization of the inhibitor. Insect Biochem Mol Biol. 2002;32(9):991–7. 12213235 10.1016/s0965-1748(02)00035-8

[13] Campos ITN, Tanaka-Azevedo AM, Tanaka AS. Identification and characterization of a novel factor XIIa inhibitor in the hematophagous insect, Triatoma infestans (Hemiptera: Reduviidae). FEBS Lett. 2004;577(3):512–6. 15556638 10.1016/j.febslet.2004.10.052

[14] Schechter I, Berger A. On the size of the active site in proteases. I. Papain. Biochem Biophys Res Commun. 1967;27(2):157–62. 6035483 10.1016/s0006-291x(67)80055-x

[15] Xu Y, Cai T-Q, Castriota G, Zhou Y, Hoos L, Jochnowitz N, et al Factor XIIa inhibition by Infestin-4: in vitro mode of action and in vivo antithrombotic benefit. Thromb Haemost. 2014;111(4):694–704. 10.1160/TH13-08-0668 24336918

[16] Campos ITN, Souza TACB, Torquato RJS, De Marco R, Tanaka-Azevedo AM, Tanaka AS, et al The Kazal-type inhibitors infestins 1 and 4 differ in specificity but are similar in three-dimensional structure. Acta Crystallogr D Biol Crystallogr. 2012;68(Pt 6):695–702. 10.1107/S0907444912009067 22683792

[17] Hojima Y, Pierce J V, Pisano JJ. Hageman factor fragment inhibitor in corn seeds: purification and characterization. Thromb Res. 1980;20(2):149–62. 6782698 10.1016/0049-3848(80)90381-3

[18] Ho SN, Hunt HD, Horton RM, Pullen JK, Pease LR. Site-directed mutagenesis by overlap extension using the polymerase chain reaction. Gene. 1989;77(1):51–9. 2744487 10.1016/0378-1119(89)90358-2

[19] Sugase K, Landes MA, Wright PE, Martinez-Yamout M. Overexpression of post-translationally modified peptides in Escherichia coli by co-expression with modifying enzymes. Protein Expr Purif. 2008;57(2):108–15. 18054500 10.1016/j.pep.2007.10.018 PMC2257981

[20] Grzesiak A, Buczek O, Petry I, Szewczuk Z, Otlewski J. Inhibition of serine proteinases from human blood clotting system by squash inhibitor mutants. Biochim Biophys Acta. 2000;1478(2):318–24. 10825543 10.1016/s0167-4838(00)00034-0

[21] Wieczorek M, Otlewski J, Cook J, Parks K, Leluk J, Wilimowska-Pelc A, et al The squash family of serine proteinase inhibitors. Amino acid sequences and association equilibrium constants of inhibitors from squash, summer squash, zucchini, and cucumber seeds. Biochem Biophys Res Commun. 1985;126(2):646–52. 3977882 10.1016/0006-291x(85)90233-5

[22] Cheng Y, Prusoff WH. Relationship between the inhibition constant (K1) and the concentration of inhibitor which causes 50 per cent inhibition (I50) of an enzymatic reaction. Biochem Pharmacol. 1973;22(23):3099–108. 4202581 10.1016/0006-2952(73)90196-2

[23] Humphrey W, Dalke A, Schulten K. VMD: visual molecular dynamics. J Mol Graph. 1996;14(1):33–8, 27–8. 8744570 10.1016/0263-7855(96)00018-5

[24] Thaimattam R, Tykarska E, Bierzynski A, Sheldrick GM, Jaskolski M. Atomic resolution structure of squash trypsin inhibitor: unexpected metal coordination. Acta Crystallogr D Biol Crystallogr. 2002;58(Pt 9):1448–61. 12198301 10.1107/S0907444902011769

[25] Phillips JC, Braun R, Wang W, Gumbart J, Tajkhorshid E, Villa E, et al Scalable molecular dynamics with NAMD. J Comput Chem. 2005;26(16):1781–802. 16222654 10.1002/jcc.20289 PMC2486339

[26] Best RB, Zhu X, Shim J, Lopes PEM, Mittal J, Feig M, et al Optimization of the additive CHARMM all-atom protein force field targeting improved sampling of the backbone φ, ψ and side-chain χ(1) and χ(2) dihedral angles. J Chem Theory Comput. 2012;8(9):3257–73. 23341755 10.1021/ct300400x PMC3549273

[27] Sadovnichy VA, Voevodin V, Opanasenko V. “Lomonosov”: Supercomputing at Moscow State University Contemporary High Performance Computing: From Petascale toward Exascale (Chapman & Hall/CRC Computational Science). Boca Raton, USA: CRC Press; 2013 p. 283–308.

[28] Wu Y, Eigenbrot C, Liang W-C, Stawicki S, Shia S, Fan B, et al Structural insight into distinct mechanisms of protease inhibition by antibodies. Proc Natl Acad Sci U S A. 2007;104(50):19784–9. 18077410 10.1073/pnas.0708251104 PMC2148376

[29] Salonen LM, Bucher C, Banner DW, Haap W, Mary J-L, Benz J, et al Cation-pi interactions at the active site of factor Xa: dramatic enhancement upon stepwise N-alkylation of ammonium ions. Angew Chem Int Ed Engl. 2009;48(4):811–4. 10.1002/anie.200804695 19101972

[30] Comeau SR, Gatchell DW, Vajda S, Camacho CJ. ClusPro: an automated docking and discrimination method for the prediction of protein complexes. Bioinformatics. 2004;20(1):45–50. 14693807 10.1093/bioinformatics/btg371

[31] London N, Raveh B, Cohen E, Fathi G, Schueler-Furman O. Rosetta FlexPepDock web server—high resolution modeling of peptide-protein interactions. Nucleic Acids Res. 2011;39(Web Server issue):W249–53. 10.1093/nar/gkr431 21622962 PMC3125795

[32] Jiménez-García B, Pons C, Fernández-Recio J. pyDockWEB: a web server for rigid-body protein-protein docking using electrostatics and desolvation scoring. Bioinformatics. 2013;29(13):1698–9. 10.1093/bioinformatics/btt262 23661696

[33] The PyMOL Molecular Graphics System. Schrodinger, LLC.; 2014.

[34] Tarandovskiy ID, Balandina AN, Kopylov KG, Konyashina NI, Kumskova MA, Panteleev MA, et al Investigation of the phenotype heterogeneity in severe hemophilia A using thromboelastography, thrombin generation, and thrombodynamics. Thromb Res. 2013;131(6):e274–80. 10.1016/j.thromres.2013.04.004 23611257

[35] Lipets E, Vlasova O, Urnova E, Margolin O, Soloveva A, Ostapushchenko O, et al Circulating contact-pathway-activating microparticles together with factors IXa and XIa induce spontaneous clotting in plasma of hematology and cardiologic patients. PLoS One. 2014;9(1):e87692 10.1371/journal.pone.0087692 24498168 PMC3909194

[36] Dashkevich NM, Ovanesov M V, Balandina AN, Karamzin SS, Shestakov PI, Soshitova NP, et al Thrombin activity propagates in space during blood coagulation as an excitation wave. Biophys J. 2012;103(10):2233–40. 10.1016/j.bpj.2012.10.011 23200057 PMC3512051

[37] Soshitova NP, Karamzin SS, Balandina AN, Fadeeva OA, Kretchetova A V, Galstian GM, et al Predicting prothrombotic tendencies in sepsis using spatial clot growth dynamics. Blood Coagul Fibrinolysis. 2012;23(6):498–507. 10.1097/MBC.0b013e328352e90e 22688554

[38] Fadeeva OA, Panteleev MA, Karamzin SS, Balandina AN, Smirnov I V, Ataullakhanov FI. Thromboplastin immobilized on polystyrene surface exhibits kinetic characteristics close to those for the native protein and activates in vitro blood coagulation similarly to thromboplastin on fibroblasts. Biochem Biokhimii͡a. 2010;75(6):734–43. 10.1134/s0006297910060088 20636265

[39] Rimphanitchayakit V, Tassanakajon A. Structure and function of invertebrate Kazal-type serine proteinase inhibitors. Dev Comp Immunol. 2010;34(4):377–86. 10.1016/j.dci.2009.12.004 19995574

[40] Apostoluk W, Otlewski J. Variability of the canonical loop conformations in serine proteinases inhibitors and other proteins. Proteins. 1998;32(4):459–74. 9726416

[41] Schneider TD, Stephens RM. Sequence logos: a new way to display consensus sequences. Nucleic Acids Res. 1990;18(20):6097–100. 2172928 10.1093/nar/18.20.6097 PMC332411

[42] Radisky ES, Koshland DE. A clogged gutter mechanism for protease inhibitors. Proc Natl Acad Sci U S A. 2002;99(16):10316–21. 12142461 10.1073/pnas.112332899 PMC124911

[43] Colman RW, Schmaier AH. Contact system: a vascular biology modulator with anticoagulant, profibrinolytic, antiadhesive, and proinflammatory attributes. Blood. 1997;90(10):3819–43. 9354649

[44] Holmes MB, Schneider DJ, Hayes MG, Sobel BE, Mann KG. Novel, bedside, tissue factor-dependent clotting assay permits improved assessment of combination antithrombotic and antiplatelet therapy. Circulation. 2000;102(17):2051–7. 11044419 10.1161/01.cir.102.17.2051

[45] Panteleev MA, Hemker HC. Global/integral assays in hemostasis diagnostics: promises, successes, problems and prospects. Thromb J. 2015;13(1):5 10.1186/s12959-014-0032-y 25642146 PMC4311466

[46] Hagedorn I, Schmidbauer S, Pleines I, Kleinschnitz C, Kronthaler U, Stoll G, et al Factor XIIa inhibitor recombinant human albumin Infestin-4 abolishes occlusive arterial thrombus formation without affecting bleeding. Circulation. 2010;121(13):1510–7. 10.1161/CIRCULATIONAHA.109.924761 20308613

