# Supplementary material for: New Infestin-4 Mutants with Increased Selectivity against Factor XIIa
Source: PLoS One. 2015 Dec 15;10(12):e0144940. doi: 10.1371/journal.pone.0144940 (PMC4684401; doi:10.1371/journal.pone.0144940)

S1 Figure. Inhibition of FXIIa by Mutant B purified with two-step chromatography. Residual amidolytic activity of fXIIa at various concentrations of Trx-fused Mutant B (0 nM; 0.25 nM; 0.5 nM; 1.0 nM; 2.0 nM; 4.0 nM; 8.0 nM). The mean ± SD values are shown (n = 2); data fitting with a hyperbola is shown with dots.

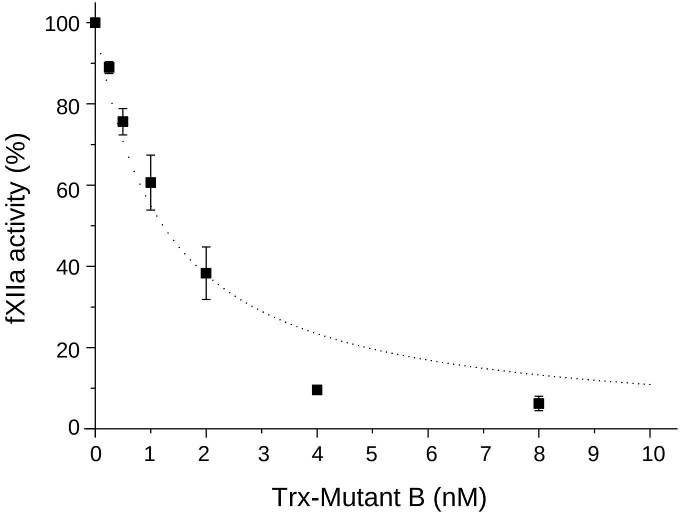

Supplement: S1 Fig — Residual amidolytic activity of fXIIa at various concentrations of Trx-fused Mutant B (0 nM; 0.25 nM; 0.5 nM; 1.0 nM; 2.0 nM; 4.0 nM; 8.0 nM). The mean ± SD values are shown (n = 2); data fitting with a hyperbola is shown with dots. (PDF) [file pone.0144940.s001.pdf]
