# Supplementary material for: New Infestin-4 Mutants with Increased Selectivity against Factor XIIa
Source: PLoS One. 2015 Dec 15;10(12):e0144940. doi: 10.1371/journal.pone.0144940 (PMC4684401; doi:10.1371/journal.pone.0144940)
Supplement: S1 Table — Restriction sites are shown in italics; overlapping complementary parts of the primers are underlined; and mutations introduced in the infestin-4 sequence are in bold. (PDF) [file pone.0144940.s002.pdf]

S1 Table. Designed primers for cloning the genes of infestin-4, its mutants and CMTI-III. Restriction sites are shown in *italics*; overlapping complementary parts of the primers are underlined; and mutations introduced in the infestin-4 sequence are in **bold**.

Primer	5'-sequence-3'
AS-KAZ-1F	tt*gctagc*gactacaaagatgatgacgataaagaggttcgtaacccatgcgctt
AS-KAZ-2R	tgccatacgttttgccatcagaaccgcacaccggaacgtagttacggaagcaagcgcatgggttacg
AS-KAZ-3F	tggcaaaacgtatggcaacccgtgcatgctcaactgtgctgcacagacgaaagtgccaggtctgaaa
AS-KAZ-4R	ttagaactgttccacgttggaacgctgacaacgaccttcgtggaccagtttcagacctggcacttt
AS-KAZ-5R	tt*aagctt*ctattagaactgttcca
MutAFor	gcttgcttccgt**cg**ct**t**cgtt**g**cggtgtgcggttctgatggc
MutARev	gcacaccg**c**aacg**a**ag**cg**acggaagcaagcgcatgggttacg
MutBFor	gcttgccg**c**aacg**a**agttacgg**gt**gtgtgcggttctgatggc
MutBRev	gcacaccg**c**aacg**a**agttacgg**gt**gcaagcgcatgggttacg
MutCFor	gcttgc**aa**ccgt**cg**ctacgttccggtgtgcggttctgatggc
MutCRev	gcacaccggaacgtag**cg**acgg**tt**gcaagcgcatgggttacg
Mut15For	gcttgc**ac**ccgt**cg**ct**t**cgtt**g**cggtgtgcggttctgatggc
Mut15Rev	gcacaccg**c**aacg**a**ag**cg**acgg**gt**gcaagcgcatgggttacg
VectorFor	cagcacatggacagcccagatctggg
VectorRev	ggtgctcgagtgcggccgc*aagctt*c
FuCMTI1 up	cgt*ccatgg*tacatcatcatcaccatcacacttacaaactgatccttaacggtaaaactctgaaaggcgttctgactatcgaagctgttgatgctgctac
FuCMTI2 low	aggttttagtagcatcgtcgtaagtccattcaccgtcaacacccaggtcgttagcgtactgtttgaacactttttctgcagtagcagcatcaacagcttc
FuCMTI3 up	cgacgatgctactaaaaccttcactgttactgaaaactacgacatcccgacctcttctggtctggttccgcgtggttctcgtgtttgcccgcgtatcctg
FuCMTI4 low	cg*gaattc*aaccgcagtaaccgtgttccaggcaaacgcattcagccaggcagtcagagtctttttgcatttcatcaggatacgcgggcaaacacg
